# Genome-wide association analysis of tan spot disease resistance in durum wheat accessions from Tunisia

**DOI:** 10.3389/fgene.2023.1231027

**Published:** 2023-10-25

**Authors:** Marwa Laribi, Rudolph Fredua-Agyeman, Sarrah Ben M’Barek, Carolina P. Sansaloni, Susanne Dreisigacker, Fernanda M. Gamba, Wided Abdedayem, Meriem Nefzaoui, Chayma Araar, Sheau-Fang Hwang, Amor H. Yahyaoui, Stephen E. Strelkov

**Affiliations:** ^1^ CRP Wheat Septoria Precision Phenotyping Platform, Tunis, Tunisia; ^2^ Department of Agricultural, Food and Nutritional Science, University of Alberta, Edmonton, AB, Canada; ^3^ Regional Field Crops Research Center of Beja (CRRGC), Beja, Tunisia; ^4^ International Maize and Wheat Improvement Center (CIMMYT), Texcoco, Mexico; ^5^ Universidad de la República Oriental del Uruguay, Paysandú, Uruguay; ^6^ Borlaug Training Foundation, Colorado State University, Fort Collins, CO, United States

**Keywords:** disease resistance, durum wheat, genetic diversity, GWAS, landraces, population structure, Tunisia

## Abstract

**Background:** Tunisia harbors a rich collection of unexploited durum wheat landraces (*Triticum durum* ssp. *durum*) that have been gradually replaced by elite cultivars since the 1970s. These landraces represent an important potential source for broadening the genetic background of elite durum wheat cultivars and for the introgression of novel genes for key traits, including disease resistance, into these cultivars.

**Methods:** In this study, single nucleotide polymorphism (SNP) markers were used to investigate the genetic diversity and population structure of a core collection of 235 durum wheat accessions consisting mainly of landraces. The high phenotypic and genetic diversity of the fungal pathogen *Pyrenophora tritici-repentis* (cause of tan spot disease of wheat) in Tunisia allowed the assessment of the accessions for tan spot resistance at the adult plant stage under field conditions over three cropping seasons. A genome-wide association study (GWAS) was performed using a 90k SNP array.

**Results:** Bayesian population structure analysis with 9191 polymorphic SNP markers classified the accessions into two groups, where groups 1 and 2 included 49.79% and 31.49% of the accessions, respectively, while the remaining 18.72% were admixtures. Principal coordinate analysis, the unweighted pair group method with arithmetic mean and the neighbor-joining method clustered the accessions into three to five groups. Analysis of molecular variance indicated that 76% of the genetic variation was among individuals and 23% was between individuals. Genome-wide association analyses identified 26 SNPs associated with tan spot resistance and explained between 8.1% to 20.2% of the phenotypic variation. The SNPs were located on chromosomes 1B (1 SNP), 2B (4 SNPs), 3A (2 SNPs), 3B (2 SNPs), 4A (2 SNPs), 4B (1 SNP), 5A (2 SNPs), 5B (4 SNPs), 6A (5 SNPs), 6B (2 SNPs), and 7B (1 SNP). Four markers, one on each of chromosomes 1B, and 5A, and two on 5B, coincided with previously reported SNPs for tan spot resistance, while the remaining SNPs were either novel markers or closely related to previously reported SNPs. Eight durum wheat accessions were identified as possible novel sources of tan spot resistance that could be introgressed into elite cultivars.

**Conclusion:** The results highlighted the significance of chromosomes 2B, 5B, and 6A as genomic regions associated with tan spot resistance.

## 1 Introduction

Durum or tetraploid wheat (*Triticum turgidum* ssp. *durum*, 2*n* = 4× = 28, AABB) is thought to have been domesticated, approximately 12,000 years ago, in the Fertile Crescent region which spans Israel, Lebanon, Syria, southeastern Turkey, Jordan, western Iran, southern Iraq, Palestine, and northern Kuwait (MacKey, 2005; Tanno and Willcox, 2006; Zohary et al., 2012; Martínez-Moreno et al., 2020). It was then introduced to the low rainfall regions of the Mediterranean basin, North Africa, Southern Europe, India and eventually the northern plains of Canada and the United States. The spread of durum wheat from the Fertile Crescent to regions all over the world is associated with human migration (Bozzini, 1988). Currently, durum wheat is considered a staple food in the Mediterranean region and North Africa, where it is grown mainly for its various end use products such as pasta, frike, couscous, and burghul, and where around 70% of worldwide durum wheat production occurs (Bonjean and Angus, 2001; MacKey, 2005; Li et al., 2013; Kabbaj et al., 2017).

Tunisia, located in North Africa and the Mediterranean basin, harbors a rich collection of durum wheat landraces, which have been gradually abandoned in favor to elite cultivars since the 1970s (Harlan, 1971; Beanjean, 2001). In Tunisia, during the 2021–2022 cropping season, wheat production was estimated at 1.2 MT over an area of 0.607 M ha (USDA, 2023). The contrasting environmental conditions of North Africa and the Mediterranean basin have led to the development of a widely diversified collection of local accessions (Mercer and Perales, 2010; Ouaja et al., 2021). Several studies reported that landraces from the secondary centers of wheat diversity such as the horn of Africa region and the Mediterranean region are genetically diverse and tolerant to both abiotic and biotic stresses (Mengistu et al., 2016; Kabbaj et al., 2017; Ouaja et al., 2020; Laribi et al., 2021; Ouaja et al., 2021; Ben M’Barek et al., 2022; Ferjaoui et al., 2022; Laribi et al., 2022b). Thus, Mediterranean landraces represent an important potential source for broadening the genetic background of elite durum wheat cultivars and for the introgression of potential novel genes for key traits into these cultivars. At present, few Tunisian farmers still sustain some of these landraces under traditional farming systems, mainly to meet their own food needs.

Nonetheless, the durum varieties grown in Tunisia remain susceptible to various fungal diseases including Septoria tritici blotch (Bel Hadj Chedli et al., 2020; Ben M’Barek et al., 2020; Ben M’Barek et al., 2023), tan spot (Kamel and Cherif, 2021) and rusts (Hammami and Gharbi, 2018), as well as abiotic stresses, particularly drought and heat (Chamekh et al., 2015). Indeed, most cultivated varieties in Tunisia were found to be susceptible to tan spot, caused by the fungus *Pyrenophora tritici-repentis*, across all wheat-growing regions of the country (Kamel and Cherif, 2021). Infection by this foliar pathogen can result in yield losses of up to 50% under favorable conditions. To date, six of the eight known races of *P. tritici-repentis* (races 2, 4, 5, 6, 7, and 8) have been identified in Tunisia. Furthermore, numerous “atypical” isolates also have been reported; these isolates cause necrosis symptoms on the susceptible genotype “Glenlea,” resembling therefore those induced by the necrotrophic effector Ptr ToxA, but lack the *ToxA* gene coding for this protein (Kamel et al., 2019; Laribi et al., 2019; 2022a).

The homothallic nature of *P. tritici-repentis*, its high genetic diversity, and the widespread adoption of conservation tillage practices that favor survival of the fungus on crop debris underscore the need to identify novel sources of tan spot resistance (Laribi et al., 2022a). Compared with hexaploid wheat (*Triticum aestivum*), only a few studies have explored tan spot resistance in tetraploid wheat. Numerous qualitative resistance genes (Friesen and Faris, 2004; Tadesse et al., 2006b; 2006a; 2007; 2010; Singh et al., 2006; 2008; 2010; Faris et al., 2013; 2020) and quantitative trait loci (QTL) conferring race-specific and non-race-specific resistance to tan spot have been identified (Faris et al., 2013; Phuke et al., 2020; Kokhmetova et al., 2021; Lozano-Ramírez et al., 2022). In addition, three susceptibility genes (*Tsn1*, *Tsc1*, and *Tsc2*) that confer sensitivity to the three necrotrophic effectors (Ptr ToxA, Ptr ToxC, Ptr ToxB) produced by *P. tritici-repentis* also have been reported (Abeysekara et al., 2010; Faris et al., 2010; 2013). The qualitative genes *tsr2*, *tsr5*, *TsrHar* and *tsr7* were found on chromosome 3B, while *tsr1*, *tsr3*, *tsr4*, *tsr6* and *TsrAri* were identified on chromosomes 5B, 3D, 3A, 2B, and 3A, respectively. A major challenge facing wheat breeders is the development of new wheat varieties that contain multiple tan spot resistance genes. Another breeding objective is to develop wheat varieties that can withstand abiotic and biotic stresses often brought about by climate change, such as increases in temperature and water scarcity, and which are expected to affect many cereal producing regions (Carew et al., 2017; Ben M’Barek and Ghaffary, 2021). Improvement in the yield and quality of wheat is another important goal for breeders, particularly with the world’s population expected to grow by 50% by 2050 (Grassini et al., 2013; FAO, 2023).

Domestication of wheat allowed the introduction of several key traits including kernel shattering resistance, larger seeds and free threshing that enabled easier harvest, better reproducibility and diversified end uses (Feldman, 2001; Charmet, 2011; Peng et al., 2011; Zohary et al., 2012; Gioia et al., 2015). Nevertheless, it is presumed that natural genetic variability in wheat was gradually lost in the process of domestication and also in the course of natural and human selection pressure for the improvement of modern cultivars (Diamond, 2002; Haudry et al., 2007; Rahman et al., 2020). Mediterranean durum wheat landraces were found to be genetically diverse, highly adapted to environmental conditions and resistant or tolerant to several abiotic and biotic stresses (Lopes et al., 2015; Ben M’Barek and Ghaffary, 2021; Laribi et al., 2021; 2022b; Ben M’Barek et al., 2022). Few studies, however, have evaluated the phenotypic and genotypic diversity of Tunisian durum wheat accessions, provided by the National Gene Bank of Tunisia (Medini et al., 2005; Robbana et al., 2019; 2021; Slim et al., 2019; Ouaja et al., 2021). These studies suggested that Tunisian durum wheat accessions are genetically diverse and well adapted to environmental conditions.

Different types of molecular markers have been employed to study the genetic diversity of wheat accessions from different agroecological origins (Poland et al., 2012; Mengistu et al., 2016; Soriano et al., 2016; Sansaloni et al., 2020). Genotyping-by-sequencing (GBS) is an advanced next-generation sequencing (NGS) approach for genotyping that is rapid, robust, high-throughput, and cost-effective method for determining the order of nucleotides in complex large sized genomes such as rice, maize, sorghum, barley, and wheat. GBS technologies including restriction-site associated DNA sequencing (RAD-seq) and DArTseq target the genomic sequence flanking restriction enzyme sites to produce a reduced representation of the genome. These two technologies have been widely used in wheat genetics (Sansaloni et al., 2011; 2020; Poland et al., 2012; Sehgal et al., 2015). DArTseq (https://www.diversityarrays.com/) technology has been applied successfully in several genomes including wheat (Sansaloni et al., 2011; 2020). This high throughput genotyping technology was first developed by Jaccoud et al. (2001) for rice, and since then has gained an increased interest (Sansaloni et al., 2020). DArTseq generates two types of markers, dominant and co-dominant, and produces less missing data compared with other GBS technologies (Sansaloni et al., 2011). It has been applied successfully in studies of genetic diversity, QTL identification, genomic selection, and GWAS (Robbana et al., 2019; Sansaloni et al., 2020).

The completion of a high-quality assembly of the genome of the durum wheat cultivar Svevo facilitated the breeding for several key traits in wheat (Maccaferri et al., 2019). The latter genome assembly provided a genome-wide account of modifications imposed by thousands of years of empirical selection and breeding and enabled fine mapping, cloning, functional analysis of genes, and accelerated the genetic improvement and the molecular-assisted selection of wheat (Maccaferri et al., 2019). Currently, with the rapid advances in next-generation sequencing (NGS) technologies, single nucleotide polymorphisms (SNPs) have become the most common type of marker used for assessment of the genetic diversity in wheat (Elshire et al., 2011; Holtz et al., 2016). The use of this type of markers in genetic studies, marker assisted selection and in genome wide association studies (GWAS), has significantly increased in the last years given their abundance in genomes, effectiveness, affordability, and potential for high-throughput screening (Gurung et al., 2011; Galagedara et al., 2020; Kokhmetova et al., 2021; Lozano-Ramírez et al., 2022).

Enhancing genetic diversity in durum wheat is an important breeding objective, while the study of this genetic diversity is important for its conservation and for understanding the evolution of this species. The exploration of wild relatives and landraces, especially within the primary and secondary centers of wheat diversity, could compensate for the loss in allelic variation as well as lead to the introgression of desirable agronomic (Lopes et al., 2015), nutritional (Cooper et al., 2001), and disease resistance (Ben M’Barek and Ghaffary, 2021; Ferjaoui et al., 2022) traits. Most Tunisian landraces, however, have not been well characterized at the molecular level or been fully exploited for cultivar development.

The objectives of this study were: 1) to evaluate the genetic diversity and population structure of a panel of Tunisian durum wheat accessions, including many landraces, by next-generation sequencing; 2) to test the collection for tan spot disease resistance at the adult plant stage under field conditions in Tunisia; and 3) to identify genomic regions associated with this resistance using GWAS. Assessment of the genetic diversity of this panel could potentially identify novel alleles for enhancing the biodiversity of durum wheat breeding materials.

## 2 Materials and methods

### 2.1 Genetic diversity of Tunisian durum wheat accessions

#### 2.1.1 Plant materials and DNA extraction

The plant materials used in this study comprised 235 durum wheat accessions obtained from the United States Department of Agriculture (USDA) National Small Grains Collection (https://www.ars.usda.gov/pacific-west-area/aberdeen-id/small-grains-and-potato-germplasm-research/docs/national-small-grains-collection/), Aberdeen, ID. The accessions included 212 landraces, 10 breeding lines, eight cultivars, and five accessions of uncertain improvement status. The complete list of accessions is provided in Supplementary Table S1.

The 235 accessions were multiplied and checked for uniformity during the 2016–2017 cropping season. Seeds of a single plant were selected for each accession, and were used for DNA extraction and then sequencing, as well as to grow plant material for field trials. Hereafter, the genotypic and phenotypic characterizations were performed for a genotype that was selected and kept distinct from the original landrace population.

Five seeds of each accession were grown under greenhouse conditions at the International Maize and Wheat Improvement Center (CIMMYT), El Batán, Mexico, for 2 weeks at 20°C with a photoperiod of 16 h light and 8 h darkness. Leaves were then harvested, frozen at −80°C, and lyophilized for 24 h. Genomic DNA was extracted in 96-well plate format from lyophilized young leaves using the cetyltrimethylammonium bromide (CTAB) method following Hoisington et al. (1994). The quality and concentration of the DNA were determined with a NanoDrop 8000 spectrophotometer V 2.1.0 (Thermo Fisher Scientific, Waltham, MA, United States).

#### 2.1.2 Genotyping

High-throughput genotyping using DArTseq™ technology (http://www.diversityarrays.com/dart-application-dartseq) was employed to generate the genomic profile of the germplasm at the Genetic Analysis Service for Agriculture (SAGA) facility at CIMMYT, El Batán, Mexico (Sansaloni et al., 2011). The DNA was prepared according to Sansaloni et al. (2011); briefly digestion and ligation reactions were performed with the restriction enzymes (RE) *PstI* and *HpaII* in order to reduce the genome complexity and generate a genomic representation of the samples. Samples were Multiplexed in 96-well microtiter plates with equimolar amounts of amplification products to run in a flow cell of a NovaSeq 6000 System (Illumina Inc., San Diego, CA). Successfully amplified fragments were sequenced to generate approximately 500,000 unique reads per sample. The FASTQ files were filtered for quality with a Phred quality score of 30 as the threshold, representing a base call accuracy of 90% for ≥50% of the bases. An additional filter was applied on the barcode sequences using a Phred quality score of 10, which represented a base call accuracy of 99.9% for ≥75% of the bases. SNP calling was performed using DArTsoft 14 (Diversity Arrays Technology, Bruce, Australia).

#### 2.1.3 SNP marker filtering

Accessions missing >20% of the genotype data were removed by filtering. In addition, the markers were filtered for minor allele frequency (MAF) >5% or >7.5% as per software requirements. In the case of filtering at MAF >5%, 9191 SNP markers were retained to determine polymorphic information content (PIC) and gene diversity (GD) with PowerMarker 3.25 (Liu and Muse, 2005). The same 9,191 markers were used to determine the population structure and conduct cluster analyses with STRUCTURE v2.3.4 (Pritchard et al., 2000) and TASSEL 5 v5.2.2.5 (Bradbury et al., 2007), respectively. The maximum number of columns in Excel (Microsoft, Toronto, ON) is 16,384, and hence GenAlEx 6.503 (Peakall and Smouse, 2006; 2012) cannot accommodate all 9,191(×2) SNP markers. Therefore, a MAF >7.5% was used to filter the markers, leaving 7,654 markers for determination of the genetic diversity within and among the subpopulations. Five thousand and seventy (5,070) of the retained SNP markers, which could be positioned on the A-genome (2,436) or B-genome (2,634), were used for linkage disequilibrium (LD) analyses. The remaining 128 SNP markers on scaffolds and 3,993 SNP markers not assigned to any of the genomes were not used for the analysis (Supplementary Table S2).

#### 2.1.4 Linkage disequilibrium

Linkage disequilibrium was calculated with TASSEL 5 v5.2.2.5 (Bradbury et al., 2007); it was measured as the allele frequency correlation (*r* ^2^) for all pairwise SNP comparisons on each chromosome and subsequently the chromosome and genome specific mean values were estimated. Inter-chromosomic LD (unlinked loci) was estimated over the whole genome. The significance of pairwise marker *r* ^2^-values was determined by calculating the Chi-square (χ^2^) statistic for each SNP pair according to Zhou et al. (2012), except that a threshold *p* < 0.001 was used to assess the level of significance. The PROC GPLOT procedure in SAS v. 9.4 (SAS Institute, Cary, NC) was used to generate LD plots of the *r* ^2^-values of pairs of markers with *p* < 0.001 vs. physical map distance (in Mb) for each chromosome. The data points were then fitted with a solid curve using the PROC TRANSREG function in SAS v. 9.4. Background linkage disequilibrium (BLD) was calculated as the *r* ^2^-values for unlinked markers that exceeded 95% (95th percentile) of the data set, following Breseghello and Sorrells (2006). The average extent of LD of each chromosome was estimated by projection of the intersection between the fitted curve and the *r* ^2^ threshold line onto the physical distance axis (Breseghello and Sorrells, 2006; Bellucci et al., 2015). The linkage disequilibrium (LD) decay pattern analysis was performed according to the Hill and Weir function [Hill and Weir, 1988, as described by Mengistu et al. (2016), Woldeyohannes et al. (2022)].

#### 2.1.5 Population structure

The genetic structure of the 235 durum wheat accessions was investigated using a Markov Chain Monte Carlo (MCMC) algorithm implemented in the population-genetic software *STRUCTURE* v2.3.4 (Pritchard et al., 2000). The admixture and allele frequency correlated models were used to determine the number of clusters (*K*) ranging from 1 to 10 with 10 itineration each and without any prior information on the origin of the accessions. The analysis was run with a burn-in length of 100,000 iterations and MCMC run length of 100,000 permutations. The optimal number of clusters (*K*) was determined via the ΔK method using structure harvester (Evanno et al., 2005; Earl and VonHoldt, 2012). Individual accessions were assigned to a sub-population if the probability of membership was ≥0.70 (Kumar et al., 2020; Aleksandrov et al., 2021).

The genetic and similarity distance matrices within and among the subpopulations and 235 accessions were calculated using GenAlEx 6.503 (Peakall and Smouse, 2006; 2012). The analysis of molecular variance (AMOVA) among and within accessions and populations and their level of statistical significance was assessed with GenAlEx 6.503 based on 10,000 permutations (Excoffier et al., 1992). In addition, patterns in the population were inferred or visualized by Principal Coordinate Analysis (PCoA) was also conducted and visualized for identified clusters (Patterson et al., 2006).

Genetic diversity among the 235 durum wheat accessions was determined using the unweighted pair group method with arithmetic mean (UPGMA) based on genetic distances and the neighbor joining (NJ) method generated with TASSEL 5 v5.2.2.5 (Bradbury et al., 2007). The latter software was used to conduct principal components analysis (PCA) based on genetic distances among accessions; principal components were generated using the covariance method and eigenvalues were generated as a measure of the proportion of variation explained by each of the principal components (Bradbury et al., 2007).

#### 2.1.6 Allele frequency based on population structure analysis

To investigate the genetics of the panel, the 235 durum wheat accessions were assigned to 18 populations based on their common names in Tunisia (Supplementary Table S1). For instance, there were six different landraces all with the name Adjini. However, these may have been collected from different geographic locations and hence may be ecotypes. In addition, farmers from different locations may assign a name that does not reflect the pedigree. Accessions with the same name were grouped together to determine their relatedness. The populations obtained comprised Adjini (6 accessions), Agili (19), Arbi (5), Azizi (7), Bidi (10), Biskri (15), Chili (8), Derbessi (7), Frigui (10), Hamira (18), Jenah Khetifah (11), Mahmoudi 17), Medea (12), MG (14), other-cvs (38), Sbei (6), Souri (12), and Unassigned Genotypes (20 accessions) (Supplementary Table S1). In addition, along with the 18 populations assigned based on common name, the subpopulations assigned following selection of the optimal *K*, were used for the AMOVA and PCoA analysis, as well as to calculate the proportion of polymorphic loci (*%P*), the number of alleles (*Na*), number of effective alleles (*Ne*), observed heterozygosity (*Ho*), diversity index (*h*), unbiased diversity index (*uh*), and Shannon’s information index (*I*) with GeneAlEx 6.503 (Peakall and Smouse, 2006). The genetic differentiation (*F*_*ST*_) was calculated at 1,000 random permutations across all loci as a measure of genetic divergence between populations (Nei, 1977). The gene flow (*Nm*) among populations was calculated based on *F*_*ST*_ using GeneAlEx 6.503 (Peakall and Smouse, 2006; 2012).

### 2.2 Genome-wide association study

#### 2.2.1 Tan spot disease evaluation

##### 2.2.1.1 Field experiments layout

The 235 durum wheat accessions were evaluated for resistance to tan spot at the adult plant stage under field conditions at the CRP Wheat Septoria Precision Phenotyping Platform, Kodia Experimental Station (36°32′51.89 N, 9°0′40.73 E), National Institute of Field Crops, Bou Salem, Tunisia. This Station is considered a very prevalent hot spot site for tan spot disease in most years (Laribi et al., 2021; 2022b). Field evaluations were carried out during the 2017–2018, 2018–2019 and 2021–2022 cropping seasons. Field experiments were conducted in an augmented design with unreplicated entries and replicated checks. Three local check durum genotypes were included in each block, consisting of the tan spot-susceptible cv. “Nasr,” the moderately susceptible “Karim,” and the resistant cv. “Salim” (Laribi et al., 2021; 2022b). Plots were irrigated to create favorable conditions for disease development and standard wheat agronomic practices were carried out to maintain the crops.

##### 2.2.1.2 Phenotyping

The field evaluations were carried out under natural inoculum conditions in 2017–2018, while in 2018–2019 and 2021–2022, wheat stubble infected with *P. tritici-repentis* and collected from previous cropping seasons was added as an inoculum source as described by Laribi et al. (2022b). Infected leaves and debris were collected and the presence of *Ptr* was confirmed (Laribi et al., 2019; 2021; 2022b). The occurence of races 2, 5, 6, 7, and 8 was reported in the experimental station (Laribi et al., 2019; 2021). All accessions were evaluated for disease resistance under field conditions at three consecutive time-points at the adult stage (Z55) (Zadoks et al., 1974) with a 7–10 days interval between each evaluation over the 3 years of trials. Disease progression was estimated by measuring tan spot incidence and severity based on a double-digit scale (00–99) (Saari and Prescott, 1975). The area under the disease progress curve (AUDPC) and relative area under the disease progress curve (rAUDPC) were calculated according to Simko and Piepho (2012), where “Nasr” was deployed as the susceptible check of the corresponding trial. Accessions were classified using rAUDPC values as follows: <0.5 resistant (R); 0.5–0.6 moderately resistant (MR); 0.6–0.7 moderately susceptible (MS); and >0.7 susceptible (S) (Ben M’Barek et al., 2022; Laribi et al., 2022b).

#### 2.2.2 Genome-wide association analysis

Due to germination issues and/or high levels of stripe rust infection, tan spot disease data was obtained only for 160 of the 235 accessions over the 3 years. In the case of the SNP markers, 4,975 of 5,070 markers located on the A and B genomes were used after further filtering for the 160 accessions at MAF >5% and genotypes missing >20% data. The GWAS was conducted with TASSEL 5 v5.2.2.5 (Bradbury et al., 2007) using the data from the 4975 SNP markers and rAUDPC values as well as the Best Linear Unbiased Predictors (BLUPs) scores for disease severity at the adult plant stage. A mixed linear model (MLM) was applied in which principal component analysis (PCA) was a fixed variate and kinship (*K*) was random. Ten additional models were tested with the general linear models (GLM) and mixed linear models (MLM) procedures implemented in TASSEL 5v 5.2.2.5 (Bradbury et al., 2007). The GLM comprised the MDS-only (multidimensional scaling), Q-only (population structure), PCA-only (Principal Component Analysis), K-only (kinship) and D-only (Distance matrix) models. The MLM comprised the MDS + K, Q + K, MDS + D, Q + D and PCA + D models (Wang et al., 2012; Li et al., 2014; Qu et al., 2017; Coan et al., 2018).

The pairwise kinship matrix was calculated with TASSEL 5v 5.2.2.5 and the kinship heatmap was plotted using the heatmap function in R v. 4.2.2 [R Core Team (2023)]. PCA was conducted with Tassel and visualized using the package ggfortify in R software (Tang et al., 2016). Quantile-Quantile (Q-Q) plots, which plot the −log10 *p*-value of the test of association (observed) with that expected given the null hypothesis of no marker-trait associations, were obtained for each model and the results of the GWAS were presented as Manhattan plots using the package qqman in R v. 4.2.2 [R Core Team (2023)] (Turner, 2018). A false-discovery rate (FDR) was used to assess the significance of the *p*-value (<0.05).

#### 2.2.3 Candidate gene analysis

The SNP markers found to be associated with tan spot resistance were used to search the GenBank Ensembl genome browser (https://plants.ensembl.org/, accessed on 2 February 2022) with the BLASTN tool. Marker sequences were realigned (BLASTN) to the *Triticum turgidum* (Svevo. v1 RefSeq Rel. 1.0) and *Triticum aestivum* (IWGSC) reference sequences. The physical positions on the reference genomes and sequence reads of SNPs were provided by SAGA. When a sequence containing a SNP did not align to a coding region, we report the most closely related gene (s) within 1,000 bp upstream and downstream (Juliana et al., 2018).

### 2.3 Statistical analysis

Significant differences between the means of the parameters (pairwise and overall) were established by Fisher’s protected least significant difference (LSD) test (*p* ≤ 0.05) in SAS v. 9.4 (SAS Institute, Inc.). The coefficient of correlation between variables (adult reactions over the different trials) was determined with the “cor.test” function in the R package “stats” (R Foundation for Statistical Computing [R Core Team (2023)], while the analysis of variance (ANOVA) was performed with the R package “stats.” The broad sense heritability (*h* ^*2*^) and Best Linear Unbiased Predictors (BLUPs) were calculated for the combinations of environments that were managed the same way, as well as for all three trials with the R package “inti.” *h* ^*2*^ was calculated using the formula *h* ^*2*^ *= Vg*/(*Vg + Verr*/*r*), where *Vg* is the genotypic variance, *Verr* is the error variance, and *r* = the number of replications (Kokhmetova et al., 2021).

## 3 Results

### 3.1 Genetic diversity of Tunisian durum wheat accessions

#### 3.1.1 Distribution and physical location of polymorphic SNPs

Paired-end reads were mapped to the wheat “Svevo cv” reference genome with Burrows-Wheeler Aligner (V. 0.7.8) (Li and Durbin, 2009; Maccaferri et al., 2019). The reference genome assembly was made available by the International Wheat Genome Sequencing Consortium and GrainGenes (RefSeq Rel. 1.0) in 2019. All SNPs were mapped to Durum Wheat (cv. Svevo) RefSeq Rel. 1.0. A total of 68,539 SNP markers were used to screen the 235 durum wheat accessions. This comprised 17,408 SNP markers on the A-genome, 19,828 markers on the B-genome, and 1,312 markers on scaffolds (UN); the remaining 29,991 markers were not assigned (NA) to any of the durum wheat genomes (Supplementary Table S2).

After filtering, 9,191 of the 68,539 were retained. These SNP markers were well distributed across all 14 chromosomes of durum wheat and comprised 2,436 A-genome, 2,634 B-genome and 4,121 non-assigned markers (Table 1). The highest number of markers was observed on chromosomes 2B (539) and 7A (535), while the lowest number was observed on chromosomes 4B (219) and 6A (239). Across the seven homoeologous sets of chromosomes, the SNP coverage ranged from 475 on chromosomes 4A and 4B to 952 on chromosomes 2A and 2B.

**TABLE 1:** Distribution and diversity index of 9,191 single nucleotide polymorphism (SNP) markers in a set of 235 durum wheat (*Triticum durum*) accessions.

A genome				
Chromosome	No. of SNP markers	No. of polymorphic markers	Gene diversity	PIC^a^
1A	289	222	0.311	0.253
2A	413	326	0.291	0.238
3A	341	271	0.302	0.247
4A	256	201	0.299	0.246
5A	363	275	0.312	0.254
6A	239	185	0.494	0.372
7A	535	397	0.260	0.218
Subtotal/mean	2,436	1877	0.324	0.261
B Genome				
1B	341	271	0.306	0.248
2B	539	424	0.317	0.258
3B	402	330	0.303	0.248
4B	219	176	0.299	0.245
5B	375	292	0.301	0.246
6B	381	314	0.315	0.255
7B	377	303	0.290	0.238
Subtotal/mean	2,634	2,110	0.304	0.248
Homoeologous				
1	630	493	0.309	0.251
2	952	750	0.304	0.248
3	743	601	0.302	0.247
4	475	377	0.299	0.245
5	738	567	0.307	0.250
6	620	499	0.405	0.314
7	912	700	0.275	0.228
Subtotal/mean	5,070	3,987	0.314	0.255
Unassigned				
Subtotal/mean	4,121	3,768	0.294	0.242
Total A + B + Unassigned	9,191	7,755	0.304	0.249

^a^ PIC, polymorphic information content.

**FIGURE 1:**
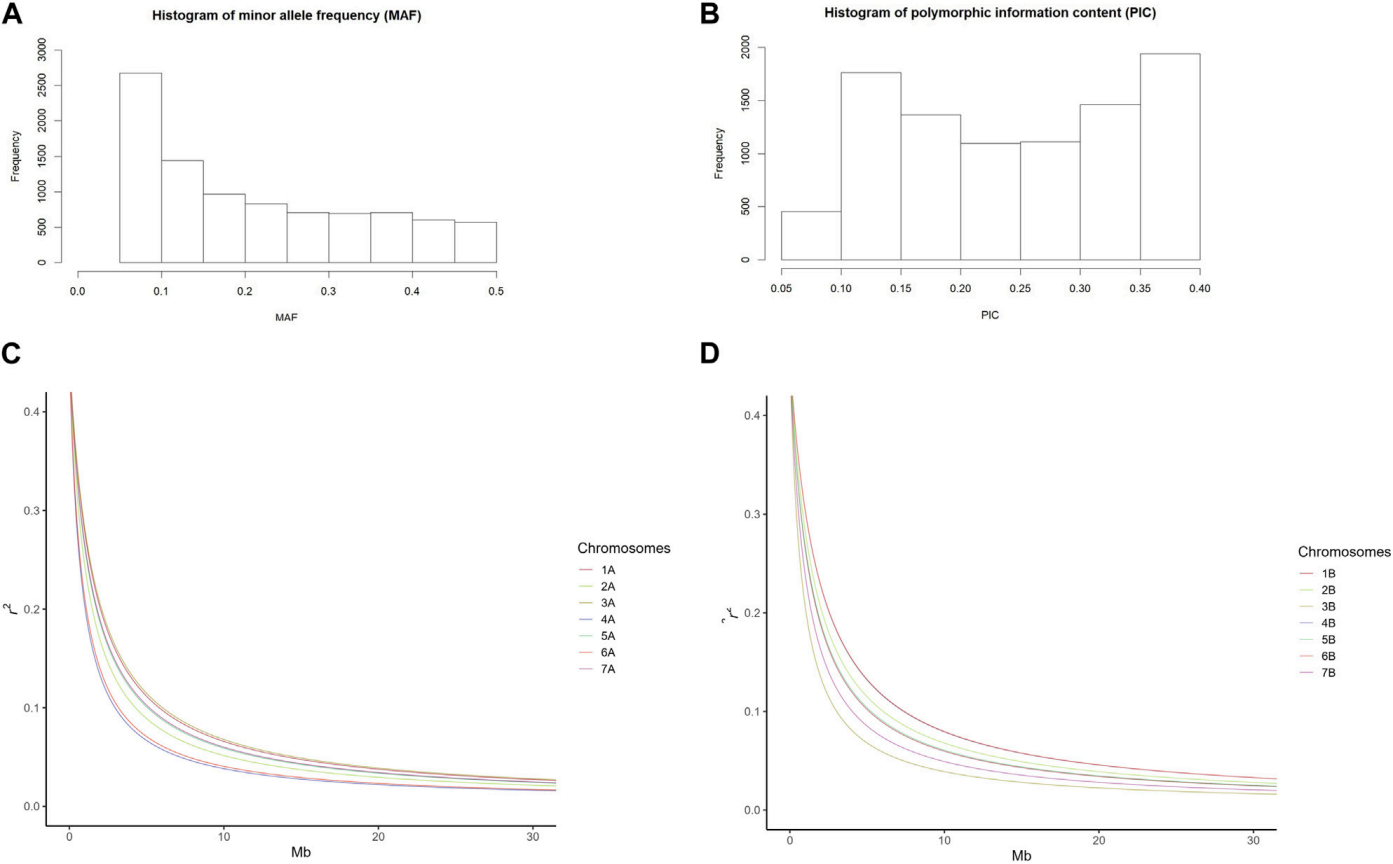
Frequency distribution of **(A)** minor allele frequency (MAF) and **(B)** polymorphic information content (PIC) of 9191 SNP markers. **(C,D)** Linkage disequilibrium (LD) decay pattern according to the Hill and Weir function; **(C)** Chromosome-specific LD decay on genome A and **(D)** Chromosome-specific LD decay on genome B.

The mean of polymorphism information content (PIC) of the SNP markers in this study was 0.246. The majority of MAFs was between 0.05 and 0.2 with a mean of 0.211 (Figures 1A, B). Nei’s genetic diversity ranged from 0.275 to 0.307 (Table 1). The genetic diversity in the A-genome was comparable with that of the B-genome (Nei’s gene diversity and PIC values of 0.324 and 0.261 for the A-genome and 0.304 and 0.248 for the B-genome, respectively) (Table 1). In the A-genome, chromosome 6A had the greatest genetic diversity (Nei’s = 0.494; PIC = 0.372) and chromosome 7A the lowest (Nei’s = 0.260; PIC = 0.218) (Table 1). In the B-genome, genetic diversity was the lowest in chromosome 7B and highest in chromosome 2B (Nei’s = 0.290; PIC = 0.238) (Table 1). Nei’s genetic diversity and PIC values across homoeologous pairs of chromosomes ranged from 0.299 to 0.405 and from 0.228 to 0.314, respectively (Table 1). Genetic diversity in the homoeologous chromosomes 6A and 6B (Nei’s = 0.405) was significantly greater compared with the other homoeologous pairs (1A/1B, 2A/2B, 3A/3B, 4A/4B, 5A/5B and 7A/7B).

#### 3.1.2 Linkage disequilibrium

The LD decay of the A- and B-genome markers covered 10 Gb of 12 Gb of the durum wheat genome, therefore representing a good coverage. The A-genome covered 4.8 Gb while the B-genome covered 5.1 Gb. The average of the squared allele correlation LD (*r* ^2^) for the A-genome, B-genome, and A + B genomes was calculated to be 0.1802, 0.1648, and 0.1723, respectively (Supplementary Table S2). About 33.6% (A-genome 34.2% + B-genome 33.4%) of the total intra-chromosomal SNP pairs were significant (*p* < 0.001). The average *r* ^2^ value ranged from 0.1483 to 0.2159 with an average of 0.1802 for the A-genome and from 0.1506 to 0.1803 with an average of 0.1648 for the B-genome. The average *r* ^*2*^ value for the A + B genomes was 0.1723 (Supplementary Table S2). The average extent of LD decay for the 14 chromosomes ranged from 12.5 to 23.8 Mb (Table 2; Supplementary Figure S1), with a mean of 14.6 Mb for the A-genome, 16.6 for the B-genome, and 15.5 Mb for the A + B genomes (Supplementary Table S2, Supplementary Figure S1). The ranges for the A- and B-genome chromosomes were 12.5–23.2 Mb and 13.8–23.8 Mb, respectively (Supplementary Table S2).

**TABLE 2:** Analysis of molecular variance (AMOVA).

Source	Df^a^	Sum squares	Mean squares	Estimated variance	Variation (%)
AMOVA of the structure groups					
Among groups	2	10484.967	5242.484	25.224	2
Within groups	467	737773.144	1579.814	1579.814	98
AMOVA of the 18 durum wheat populations based on common name					
Among Populations	17	77094.583	4534.975	119.187	7
Within Populations	452	671163.528	1484.875	1484.875	93
Among individuals	217	585349.528	2697.463	1166.149	73
Within individuals	235	85814.000	365.166	365.166	23

^a^ Df, degrees of freedom.

Further analyses indicated that homoeologous chromosomes of genomes A and B showed different LD decay patterns (Figures 1C, D). The LD halving distance of chromosomes 1, 3, and 7 exhibited a much slower decay in genome A (Figure 1C) while the LD halving distance of chromosomes 2, 4, and 6 showed a much slower decay in genome B (Figure 1D). The LD decay patterns of chromosomes 5A and 5B were very similar (Figures 1C, D). These different LD decay patterns confirm the genetic differences between the A and B genomes.

Additionally, the LD half decay was estimated for all 14 chromosomes on a defined *r* ^*2*^ of 0.1. It ranged from 2.9 Mb for chromosome 4A to 7.3 Mb for chromosome 6B (Supplementary Table S2). The average LD half decay was equal to 4.6 and 5.4 Mb for genome A and genome B, respectively (Supplementary Table S2). The average LD half decay was equal to 5 Mb for both A and B genomes (Supplementary Table S2).

#### 3.1.3 Bayesian population structure analysis

**FIGURE 2:**
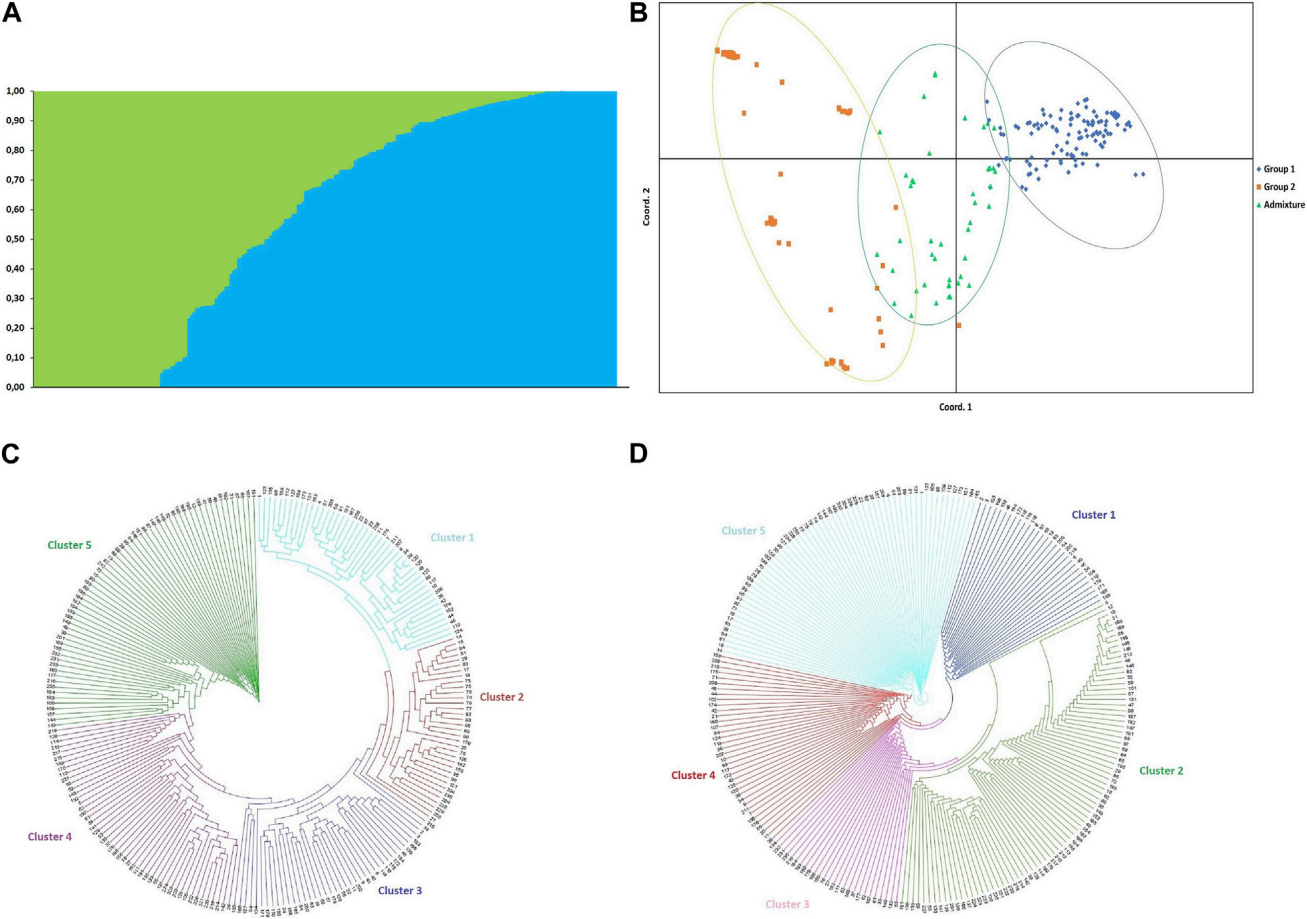
**(A)** Bayesian clustering of 235 durum wheat (*Triticum durum*) accessions from Tunisia based on 9,191 SNP markers sorted by Q; the optimal value of *K*, determined by the method of Evanno et al. (2005) with populations unassigned suggested that the 235 accessions could be placed into two groups (*K* = 2) **(B)** Principal coordinates analysis (PCoA) based on structure grouping. **(C)** Neighbour joining (NJ) and **(D)** unweighted pair group method with arithmetic mean (UPGMA) analyses with 9,191 SNP markers grouped the 235 durum wheat accessions from Tunisia into five clusters.

The optimal value of *K*, determined by the method of Evanno et al. (2005) with unassigned populations, suggested the presence of two subpopulations in the accessions. Based on a probability of membership of ≥0.70, 117 of the accessions (49.79%) were placed in group 1, 74 (31.49%) were placed in group 2, and 44 accessions (18.72%) were classified as admixture (Supplementary Table S1; Figure 2A).

#### 3.1.4 Allelic patterns and genetic diversity indices

Allelic patterns and genetic diversity summary statistics determined with GenALEx at any given locus or averaged across the 7,654 SNP loci groups assigned based on structure results and populations assigned based on common name are presented in Supplementary Tables S3, S4 and Supplementary Figures S2A–D.

For groups based on structure, the overall Shannon’s information index (*I*) Observed Heterozygosity (*Ho*) and Unbiased Expected Heterozygosity (*uHe*) were equal to 0.680 (±0.001), 0.096 (±0.001) and 0.407 (±0.001), respectively (Supplementary Table S3).

For populations assigned based on common name, the proportion of polymorphic loci (%*P*) ranged from 74.0% to 99.7% for the populations Arbi and Unassigned Genotypes, respectively, with an overall average of 92.0% (Supplementary Table S4). The Shannon’s information index (I) was significantly higher in the populations Mahmoudi (0.756 ± 0.003), Biskri (0.693 ± 0.003) and Hamira (0.659 ± 0.003) compared with the other populations. The overall Shannon’s information index (I) was equal to 0.519 (±0.001). The diversity of the SNP markers expressed as the PIC ranged from 0.246 to 0.370 with an average of 0.313 (Supplementary Figure S2B). The MAF ranged from 0.185 to 0.335 with an average of 0.272. The Gene Diversity (D) ranged from 0.266 to 0.406 with an average of 0.338. The populations Adjini, Azizi, Biskri, Chili, Derbessi, Mahmoudi, MG and Sbei had PIC, MAF and D values lower than the average, while the remaining populations Agili, Arbi, Bidi, Frigui, Hamira, Medea, other-cvs, Souri and Unassigned Genotypes had values higher than the average for all of these genetic indices (Supplementary Table S5).

#### 3.1.5 Genetic differentiation among the groups

The overall fixation statistics index (*F*_*ST*_) for groups based on structure was 0.010 (*p* ≤ 0.001). The *F*_*ST*_ values ranged from 0.004 to 0.019; the main difference was found between group 1 and the admixture (*F*_*ST*_ = 0.019), followed by group 1 and group 2, and group 2 and the admixture (*F*_*ST*_ = 0.008 and 0.004, respectively). The overall gene flow (*Nm*) was equal to 23.696. Pairwise comparisons indicated that gene flow (*Nm*) was highest between group 2 and the admixture (*Nm* = 63.600) followed by group 2 and group 1 (*Nm* = 31.620), while the lowest gene flow was found between group 1 and the admixture (*Nm* = 13.112).

The overall fixation statistics index (*F*_*ST*_) for populations based on common name was equal to 0.045 (*p* = 0.001). The *F*_*ST*_ values for all 154 pairwise combinations of all 18 populations ranged from 0.0 to 0.124. Pairwise comparisons of population differentiation based on *F*_*ST*_ are presented in Supplementary Table S6. The pairwise comparisons of gene flow (*Nm*) values ranged from 1.943 (for Adjini and Chili) to 97.422 (for Arbi and Sbei).

AMOVA of groups assigned based on structure results indicated that 76% of the variance was among individuals, 23% was between individuals, and that 98% of the variance was among populations while 2% of the variation occurred among populations. This suggested only minor differences between groups 1, 2 and the admixture. The principal coordinate analysis (PCoA) based on the 7,654 SNP markers clustered the 235 accessions into three heterogeneous subgroups (Figure 2B) consistent with the *STRUCTURE* grouping using the first (PCoA1 ≈ 12.52% of genetic variance) and second (PCoA2 ≈ 4.64% of genetic variance) principal coordinates. AMOVA of populations assigned based on common name indicated that 73% of variance was among individuals, 23% was between individuals, and that 93% of the variation occurred within the 18 populations while 7% was among populations (Table 3).

**TABLE 3:** Analysis of variance (ANOVA) of the relative area under the disease progress curve on 235 durum wheat accessions during three cropping seasons (2017–2018, 2018–2019 and 2021–2022).

Source of variation	Df^a^	Sum of squares	Mean of squares	F value	Pr (>F)
Year	2	0.302	0.15090	5.52	0.00428**
Residuals	456	12.465	0.02734		
Genotype	159	5.288	0.03326	1.33	0.0183*
Residuals	299	7.479	0.02501		

^a^ Df, degrees of freedom; Significance codes: **0.001, *0.05.

#### 3.1.6 Cluster analysis

The neighbor-joining (NJ) and unweighted pair grouped method with arithmetic mean (UPGMA) clustering based on the 9,191 SNP markers grouped the 235 accessions into five major branches (Figures 2C, D). For the NJ clustering, clusters 1, 2, 3, 4, and 5 included 45, 34, 41, 39, and 76 accessions, respectively. The Adjini accessions were distributed equally between clusters 1, 3, and 5 (two accessions each). Most of the Biskri, Mahmoudi and other-cvs accessions were grouped in cluster 5 [10 (66.7%), 14 (82.4%), and 13 (34.2%) accessions, respectively (Figures 2C, D)]. For the UPGMA clustering, clusters 1, 2, 3, 4, and 5 included 30, 81, 26, 37, and 61 accessions, respectively. In the case of the population Azizi, 28.6% of the accessions were grouped in cluster 2, while 71.4% were in cluster 5. Most Agili accessions (42.1%) grouped in cluster 4, while 53.3% of the Biskri accessions, 62.5% of the Chili accessions, 76.5% of the Mahmoudi accessions, and 50% of the MG accessions grouped in cluster 2. Cluster 5 included 58.3% of the Medea accessions, 50.0% of the Frigui accessions, and 71.4% of the Azizi accessions. In general, there was no clear clustering of the accessions based on their pedigree using either the NJ or UPGMA method (Figures 2C, D). Interestingly, accessions in cluster 2 of the UPGMA analysis were all included in either cluster 4 or cluster 5 of the NJ analysis, suggesting that the latter two clusters could be combined, reducing the number of NJ clusters from five to four. Overall, the three multivariate analyses (PCoA + NJ + UPGMA) suggested the existence of three to five groups in the durum wheat accessions, although correlations with their pedigree were low.

Cluster 3 of the NJ tree included the lowest number of populations (10), including Adjini, Agili, Bidi, Biskri, Hamira, Jenah_Khetifah, Mahmoudi, other-cvs, Souri, and Unassigned Genotypes. Similarly, cluster 1 of the UPGMA tree included the lowest number of populations (10), including Adjini, Agili, Bidi, Biskri, Chili, Hamira, Jenah_Khetifah, Mahmoudi, other-cvs, Souri, and Unassigned Genotypes. When comparing the Bayesian cluster analysis with the NJ clustering, the Bayesian group 1 included 38.5%, 29.1%, 26.5%, and 6.0% of the NJ clusters 1, 2, 3, and 5, respectively. The Bayesian group 2 included 77.0% and 23.0% of NJ clusters 5 and 4, respectively, while the admixture included 50.0%, 27.3% and 22.7% of the NJ clusters 4, 5, and 3, respectively. In contrast, all of Bayesian group 2 was found in the UPGMA cluster 1, while Bayesian group 1 included 50.4%, 30.8%, and 17.8% of the UPGMA clusters 5, 4, and 1, respectively. Over 50% of the accessions in the populations Adjini, Agili, Azizi, Frigui, Hamira, Jenah-Khetifah, Medea, other-cvs, and Unassigned Genotypes clustered in Bayesian group 1, while >50% of the accessions in the populations Arbi, Biskri, Chili, and Sbei clustered in Bayesian group 2.

### 3.2 Genome-wide association study

#### 3.2.1 Population kinship

**FIGURE 3:**
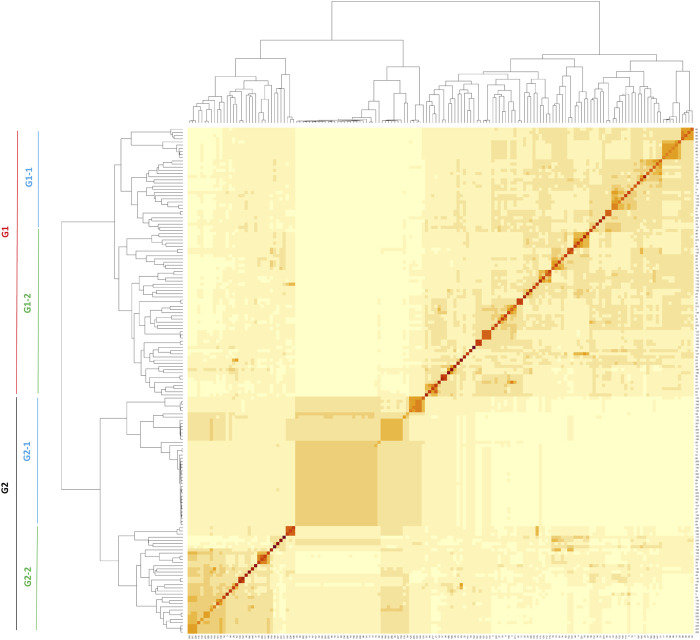
Kinship matrix of the 160 accessions used in GWAS.

A kinship heatmap plot displaying the phylogenetic relationships among the 160 accessions revealed two main groups (Figure 3). Each of the two main groups could be subdivided into two additional groups (Figure 3). The first group G1 included 52.5% of the accessions (39.3% and 60.7% in sub-groups G1-1 and G1-2, respectively) while the second group G2 included 47.5% of the accessions (55.3% and 44.7% in sub-groups G2-1 and G2-2, respectively) (Figure 3; Supplementary Table S1).

#### 3.2.2 Field resistance to tan spot

**FIGURE 4:**
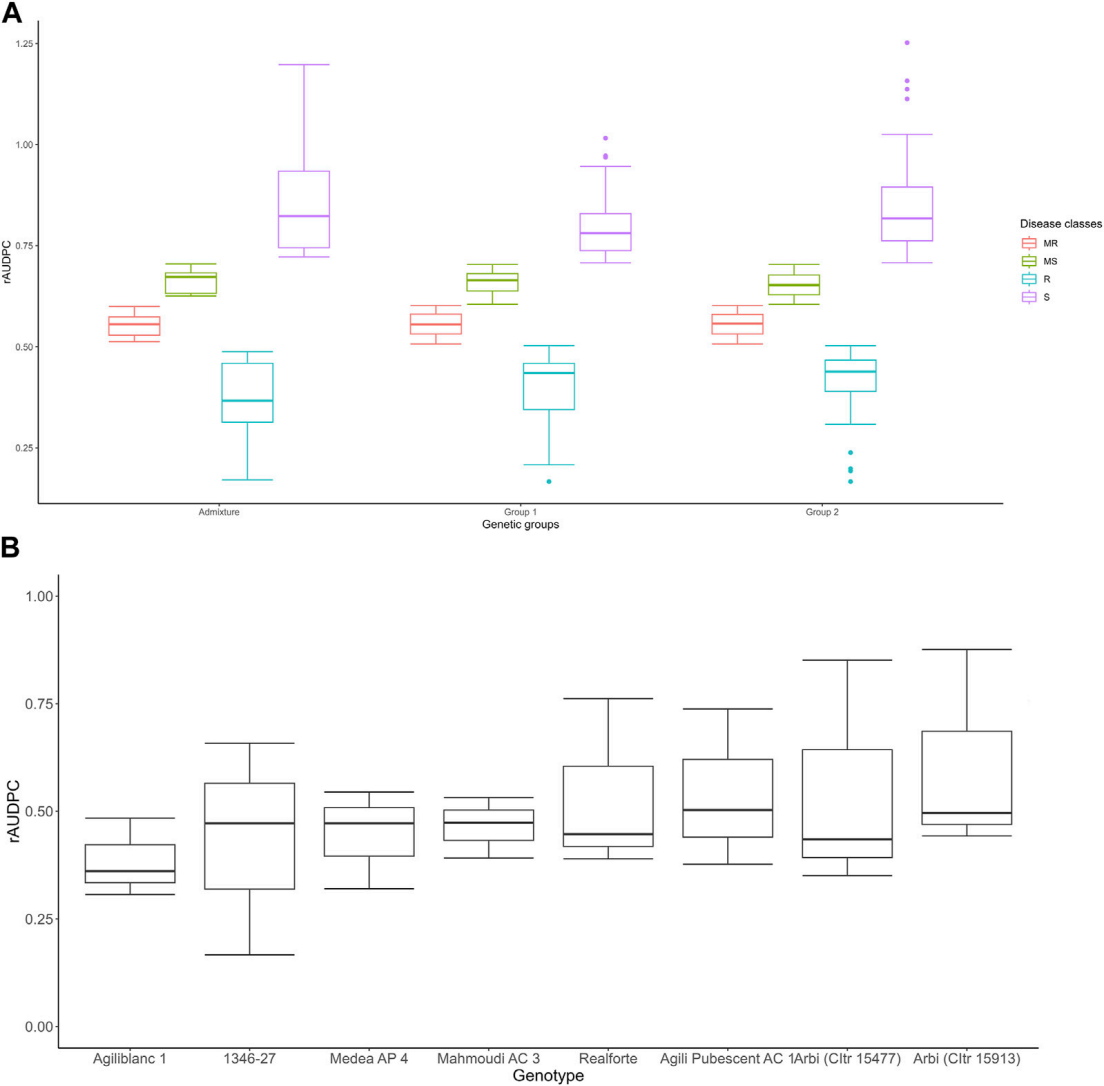
Boxplot of distribution of phenotypes in different genetic groups; the box boundaries indicate the 25th (lower) and 75th (upper) percentile, and the line within the box indicates the median value. The whiskers above and below the box represent the highest and lowest values, respectively **(A)**. Boxplot of eight accessions that showed a high level of tan spot resistance in at least at two of the three cropping seasons **(B)**.

The 160 accessions analyzed for tan spot reaction are presented in Supplementary Table S1. On average, 20%, 23%, 27%, and 30% of the accessions were resistant (R), moderately resistant (MR), moderately susceptible (MS) or susceptible (S) in the first trial under natural infection (2017–2018 cropping season) (Supplementary Figure S3). In the second trial (2018–2019), in which stubble-borne inoculum was added, 17.5%, 17.5%, 31%, and 34% of the accessions were rated R, MR, MS, and S, respectively (Supplementary Figure S3). In the third trial (2021–2022), which also included the addition of stubble-borne inoculum, 7%, 19%, 27%, and 47% of the accessions were rated as R, MR, MS, and S, respectively (Supplementary Figure S3). The distribution of different disease phenotype classes as well as their variation in different genetic groups assigned based on structure results, are displayed in Figure 4.

Based on the rAUDPC scores, eight accessions [Agili Pubescent AC 1, Medea AP 4, 1346–27, Arbi (CItr 15477), Arbi (CItr 15913), Realforte, Agili blanc 1, and Mahmoudi AC 3] showed a strong level of resistance in at least two of the three trials (Figure 4B). The Pearson correlation coefficient (*r*) between the two trials in which stubble-borne *P. tritici-repentis* inoculum was applied was highly significant (*r* = 0.34, *p* ≤ 0.001); however, no significant correlation was found between the first trial under natural infection and the two trials with inoculum added. Analysis of variance indicated a significant difference in tan spot response (*p* ≤ 0.001) among all three cropping seasons. This suggested that the genotype had a significant effect on disease response (*p* ≤ 0.05) (Table 3). The broad sense heritability (*h* ^*2*^) estimates for tan spot disease for 2018–2019 and 2021–2022 where infected wheat stubble was added as an inoculum source was equal to 0.55 while that of all three trials (natural and artificial inoculation) was equal to 0.28.

#### 3.2.3 Marker-trait associations for relative area under the disease progress curve

**FIGURE 5:**
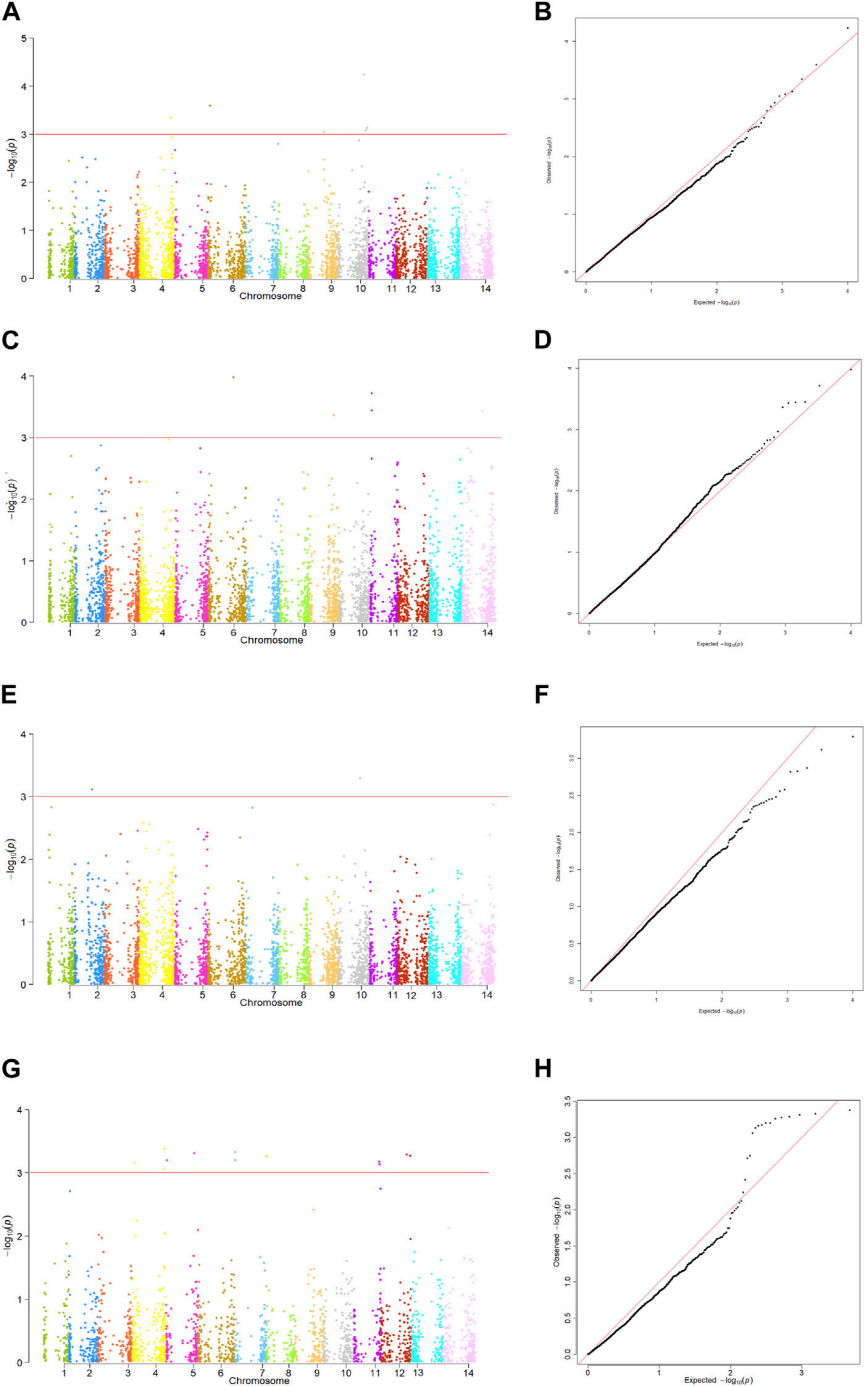
Manhattan plots and QQ plots of the MLM + PCA + K model showing significant markers associated with tan spot resistance in 160 Tunisian durum wheat (*Triticum durum*) accessions. **(A)** Manhattan plot for the rAUDPC scores in 2017–2018. **(B)** QQ plot for GWAS results of rAUDPC 2017–2018. **(C)** Manhattan plot for the rAUDPC scores in 2018–2019. **(D)** QQ plot for GWAS results of rAUDPC 2018–2019. **(E)** Manhattan plot for the rAUDPC scores in 2021–2022. **(F)** QQ plot for GWAS results of rAUDPC 2021–2022. **(G)** Manhattan plot for the GWAS with BLUP of both inoculated trials. **(H)** QQ plot of BLUP of both inoculated trials Chromosomes are identified as follows: 1 = 1A, 2 = 1B, 3 = 2A, 4 = 2B, 5 = 3A, 6 = 3B, 7 = 4A, 8 = 4B, 9 = 5A, 10 = 5B, 11= 6A, 12 = 6B, 13 = 7A, and 14 = 7B.

Based on the QQ plots, the MLM + PCA + K model was found to be the best fit for identifying genomic regions associated with tan spot resistance (data not shown). Hence, only the MLM + PCA + K model was used to determine all marker-trait associations (Figure 5).

Analysis of the rAUDPC and BLUP scores for the three trials found 26 SNPs could be associated with disease resistance. These SNPs were identified on all chromosomes with the exception of chromosomes 1A, 2A and 7A. They comprised 1, 4, 2, 2, 2, 1, 2, 4, 5, 2 and 1 SNPs on chromosomes 1B, 2B, 3A, 3B, 4A, 4B, 5A, 5B, 6A, 6B and 7B, respectively (Figure 5; Table 4). All identified SNP markers found to be associated with tan spot explained between 8.1% and 20.2% of the phenotypic variation in disease response. Six SNPs were associated with the rAUDPC scores in 2017–2018; these included one on each of chromosomes 2B, 3B, and 5A, and three on chromosome 5B. These markers explained 8.1%–12.7% of the variation. Markers ID 2271039 (5B) and ID 3064632 (3B) explained the highest percentage of the phenotypic variation (10.8%–12.7%) (Figure 5; Table 4). The markers effect size ranged from −0.16 to 0.25. The markers with the highest allele effect were located on chromosomes 3B (−0.26), 2B (0.25), and 5B (0.25). Similarly, six SNP markers were associated with the rAUDPC scores in 2018–2019; these included one on each of chromosomes 3B, 5A, and 7B, and three on chromosome 6A. These SNP markers explained 10.2%–14.0% of the phenotypic variance, with SNP ID 4989018 (6A) explaining the highest percentage followed closely by ID 5577017 (3B) that explained 13.6% (Figure 4; Table 4). The markers effect size ranged from 0.22 to 0.51. The markers with the highest allele effect were located on chromosomes 6A (0.41 and 0.51). For the rAUDPC scores obtained in 2021–2022, two SNP markers were identified; these included ID 1135724 on chromosome 5B and ID 100050780 on chromosome 1B, which explained 10.3% and 11.4% of the phenotypic variance, respectively (Figure 5; Table 4). The markers effect size ranged from 0.27 to 0.38. For the BLUP scores of the two trials with artificial inoculation, twelve SNPs were identified, which included 3, 2, 2, 1, 2, and 2 SNPs on chromosomes 2B, 3A, 4A, 4B, 6A, and 6B, respectively (Table 4; Figure 5). These markers explained 16.2%–20.2% of the variation (Table 4). Marker ID 1279775 on chromosome 2B explained the highest percentage of variation (20.2%) (Table 4). Markers identified on chromosome 2B and 3A explained 16.2%–20.2% and 17.4% of variation, respectively (Table 4). The markers effect size ranged from −0.16 to −0.29. SNPs identified on chromosome 4A explained 16.5%–19.1% of variation while the identified marker (ID 1119379) on chromosome 4B accounted for 16.5% (Table 4). Similarly, markers identified on chromosomes 6A and 6B accounted for 16.2%–18.4% and 18.1%–19.7% of the variance, respectively (Table 4).

**TABLE 4:** Markers associated with the relative area under the disease progress curve (rAUDPC) in durum wheat (*Triticum durum*) under natural tan spot (*Pyrenophora tritici-repentis*) inoculum conditions in 2017–2018 and under artificial inoculation (infected wheat stubble) 2018–2019 and 2021–2022 at −log10 *p* ≥ 3 for model MLM + PCA + K.

Trait	Marker	Genetic position on Wheat_Durum_cv. Svevo_v1 (bp)	Genetic position on Chinese spring (bp)	Genetic position on consensus map (cM)	*p*-value	Marker *R* ^*2*^	MAF	Effect
rAUDPC 2017–2018	2271039	5B|586,430,662	5B|589,677,807	5B|76.4	5.80E−05	12.7	0.397	−0.22
rAUDPC 2017–2018	3064632	3B|52,729,023	3B|47,197,513	3B|35.5	2.54E−04	10.8	0.121	−0.26
rAUDPC 2017–2018	1107872	2B|705,651,010	2B|717,692,698	2B|83.2	4.52E−04	8.5	0.085	0.25
rAUDPC 2017–2018	2262945	5B|659,693,343	5B|668,828,146	5B|120.4	7.39E−04	8.2	0.330	0.13
rAUDPC 2017–2018	1127995	5B|631,281,467	5B|635,531,780	5B|108.1	8.25E−04	8.1	0.060	0.25
rAUDPC 2017–2018	2276400	5A|349,758,849	5A|356,475,232	5A|38.3	8.87E−04	8.8	0.312	−0.16
rAUDPC 2018–2019	5577017	3B|561,192,900	3B|566,605,697	—	1.04E−04	13.6	0.223	0.31
rAUDPC 2018–2019	4989018	6A|21,182,099	6A|24,408,090	6A|32.2	1.92E−04	14.0	0.400	0.51
rAUDPC 2018–2019	990930	6A|24,685,785	6A|25,630,335	6A|287	3.54E−04	10.2	0.085	0.22
rAUDPC 2018–2019	1139857	6A|21,906,784	6A|24,845,173	6A|287	3.61E−04	11.4	0.081	0.41
rAUDPC 2018–2019	1099093	7B|453,440,752	7B|469,742,950	7B|47.2	3.68E−04	11.4	0.112	0.38
rAUDPC 2018–2019	1109903	5A|536,115,546	5A|574,195,169	5A|81.5	4.32E−04	10.5	0.060	0.27
rAUDPC 2021–2022	1135724	5B|480,124,219	5A|503,430,449	5A|56.0	5.06E−04	10.3	0.101	−0.15
rAUDPC 2021–2022	100050780	1B|390,668,089	1A|368,815,830	—	7.64E−04	11.4	0.461	−0.31
BLUP inoculated trials	1279775	2B|759,631,366	—	2B|94.8	4.19E−04	20.2	0.139	−0.29
BLUP inoculated trials	1106958	2B|53,701,140	2B|53,996,830	2B|40.7	6.84E−04	16.2	0.243	−0.29
BLUP inoculated trials	4991617	2B|734,907,676	2A|744,905,320	2B|86.0	8.71E−04	17.0	0.495	−0.17
BLUP inoculated trials	3064370	3A|646,901,140	3A|655,669,429	3A|65.7	4.84E−04	17.4	0.115	−0.29
BLUP inoculated trials	1104851	3A|17,053,466	3B|22,917,259	3B|19.1	6.31E−04	17.4	0.273	−0.29
BLUP inoculated trials	1090716	4A|15,906,034	4A|16,344,296	4A|5.9	4.69E−04	19.1	0.417	−0.29
BLUP inoculated trials	2248753	4A|16,592,100	4A|17,023,815	4A|13.1	6.31E−04	16.5	0.461	−0.29
BLUP inoculated trials	1119379	4B|9,785,826	4B|10,579,590	4B|9.2	5.45E−04	16.5	0.306	−0.29
BLUP inoculated trials	1092576	6A|579,998,288	6A|584,947,190	6A|79.1	6.71E−04	16.2	0.103	−0.29
BLUP inoculated trials	1078005	6A|588,496,558	6A|593,657,698	—	7.34E−04	18.4	0.161	−0.29
BLUP inoculated trials	10983799	6B|592,947,055	6B|616,449,494	6B|34.9	5.14E−04	19.7	0.050	−0.29
BLUP inoculated trials	1074139	6B|671,433,787	6A|604,537,957	6A|74.3	5.33E−04	18.1	0.405	−0.28

— Unknown genetic position.

All identified SNPs represent independent SNP markers with the exception of the two markers on chromosome 6A (ID 4989018 and ID 1139857) which were closely positioned and thus may represent one SNP associated with tan spot resistance. In conclusion, 25 SNP markers identified in this study could be considered as independent.

The SNP markers identified in this study were positioned on the reference genomes *Triticum aestivum* IWGSC1.0 RefSeq v1.0 (Table 4) and the consensus map (Sansaloni et al., 2020). Twenty-one of the 26 SNP markers overlapped with those mapped on the Svevo-cv v1 reference genome, whereas four showed different but homologous chromosome assignments. One SNP marker could not be located on any chromosome (Table 4). Similarly, when the 26 SNP markers were aligned to the consensus map, 20 markers overlapped with those mapped on Svevo-cv v1 reference genome, while three SNPs showed a different but homologous chromosome assignment and three markers did not have a genetic position (Table 4). Overall, alignment with the two reference genomes indicated that 11 of the 26 SNP markers identified in this study were positioned on the A-genome, while 15 markers were on the B-genome.

#### 3.2.4 Candidate genes based on annotation

Nineteen of the 26 SNP markers identified with the MLM PCA + K model occurred within annotated high-confidence gene sequences in the reference genomes *Triticum turgidum* (Svevo. v1) and *Triticum aestivum* (IWGSC) (Supplementary Table S8). Sixteen of the 19 SNP markers, with the exception of markers ID1099093, ID 3064370, and ID 1090716, were of particular interest as they overlapped with genes that code for resistance-related proteins (Supplementary Table S8). The identified candidate genes code for several proteins with significant roles in biotic stress resistance, including UDP-Glycosyltransferase, Basic helix-loop-helix (BHLH) transcription factor, zinc finger, Fatty acid metabolism regulator protein G, GDSL lipase/esterase, F-box family protein, Plant regulator RWP-RK family protein, putative Protein NLP, NAC domain protein, Glutamate receptor, BTB/POZ (zinc finger) Protein trichome birefringence, Endo-1,4-beta-xylanase, Galactose-binding-like domain superfamily, Xyloglucan fucosyltransferase, and Protein DMP (Supplementary Table S8).

When a sequence containing a SNP did not align to a coding region, we reported the closest gene (s) within 1,000 bp upstream and downstream, as well as the nearest gene that was not within this 1,000 bp upstream and downstream region. These genes are listed in Supplementary Table S8. In all, three and five candidate genes were within the 1,000 bp upstream and downstream range on the *Triticum turgidum* (Svevo. v1) and *Triticum aestivum* (IWGSC) reference genomes, respectively. In contrast, five and six candidate genes were within a few thousand basepairs of the SNP position on the *T. turgidum* (Svevo. v1) and *T. aestivum* (IWGSC) reference genomes, respectively.

## 4 Discussion

Tunisia was once the breadbasket of the Roman Empire, and at present is one of the major durum wheat producers in North Africa and the Mediterranean basin (Sadok et al., 2019; Ben M’Barek and Ghaffary, 2021). Despite the importance of durum wheat as a staple crop in Tunisia, yield is inconsistent and remains below the national potential (USDA, 2023), largely due to various abiotic and biotic challenges (Ben M’Barek and Ghaffary, 2021; Kamel and Cherif, 2021; Ben M’Barek et al., 2023). Since the 1970s, Tunisian farmers gradually abandoned landraces in favor of modern elite cultivars introduced by CIMMYT and ICARDA. The modern cultivars are susceptible to several biotic stresses including Septoria tritici blotch and tan spot, which can lead to significant yield losses and the excessive use of fungicides (Kamel and Cherif, 2021; Ben M’Barek et al., 2023). Wheat landraces are heterogeneous populations and contain a mixture of genotypes composed of diverse alleles and genotypes. The evolution of landraces over many generations for thousands of years in multiple contrasting environments and under different cultural practices, led to the development of a collection of untapped reservoirs of valuable traits for biotic and abiotic stresses (Jaradat, 2006). These landraces have encountered several evolutionary processes between different populations mainly through seed exchange, and natural and human selection (Harlan and Chapman, 1992; Nachit et al., 1995; Villa et al., 2005; Jaradat, 2006; Oliveira et al., 2014). In this context, the selection of a single plant of each accession in our study allowed the reduction of observed heterozygosity of single genotypes compared with the original landrace population. It has been reported that the genetic diversity of wheat has decreased due to the pressure from the pure-line selection applied in breeding programs and the use of a limited sources for introgression (Royo et al., 2009). Therefore, the development of new varieties from landrace populations is a critical strategy to improve modern elite cultivars and broaden their genetic background (Jaradat, 2013; Jaradat and Shahid, 2014).

### 4.1 Single nucleotide polymorphisms

Previous studies have reported that Tunisian durum wheat landraces are agro-morphologically diversified, harbor a broad range of technological properties including heavy grains and high grain-filling rates, and represent good sources of resistance to several abiotic and biotic stresses (Ayed et al., 2010; Ayadi et al., 2012; Nazco et al., 2012; Chamekh et al., 2015; Babay et al., 2019; Ouaja et al., 2020; 2021; Yacoubi et al., 2020). Most recently, genotyping-by-sequencing (GBS) has proven to be a rapid and cost-effective method to investigate germplasm diversity, population structure, and conduct genome-wide association analysis. The distribution of the markers used in this study was equal between the A- and B-genomes, with the highest number of markers on chromosomes 7A and 2B, similar to the study of Robbana et al. (2019).

Previous studies conducted on Tunisian durum wheat landraces from the National Gene Bank of Tunisia used AFLP, SSR, and DArT markers (Medini et al., 2005; Robbana et al., 2019; Slim et al., 2019). In this study, SNP markers were used as reliable and cost-effective tools to evaluate the genetic diversity and population structure of a core collection of durum wheat landraces from Tunisia provided by the USDA. Compared with the previous studies, these SNP markers were distributed equally on the A- and B-genomes. This enabled a high genome coverage and hence provided a better estimation of the genetic diversity indices and the marker-tan spot association in the GWAS studies. The PIC value was equal to 0.246, which was higher than that reported by Robbana et al. (2019) (PIC = 0.165) using 16,148 DArTseq markers. Baloch et al. (2017) reported PIC values of 0.265 and 0.302 using DArTseq and SNP markers, respectively, while Kabbaj et al. (2017) and Ren et al. (2013) reported a PIC of 0.32 and 0.188, respectively, using SNP markers. The overall mean PIC value of a collection of Ethiopian durum wheat landraces was equal to 0.203 (Alemu et al., 2020). Therefore, the PIC value obtained in this study was comparable to those reported in studies of durum wheat using bi-allelic markers such as SNPs or DArT. In contrast, the PIC value in the current study was lower than the PIC values of 0.57–0.72 previously reported with SSR markers (Medini et al., 2005; Slim et al., 2019; Ouaja et al., 2021). This was expected, since PIC values obtained with bi-allelic markers such as DArT and SNPs cannot exceed 0.50, while they can reach up to 1.0 with multi-allelic markers such as AFLP and SSRs (Hurtado et al., 2008; Semagn et al., 2014; Targońska et al., 2016). Therefore, the variability of PIC and genetic diversity between studies conducted using Tunisian durum wheat landraces may reflect the different types of markers, number of landraces, and number of genotypes per landrace (as well as their origin) in different studies. The genetic diversity in this study ranged from 0.266 to 0.406 with an average of 0.338, which was higher than the 0.275 to 0.307 reported by Robbana et al. (2019). The genetic diversity of a collection of Ethiopian durum wheat landraces varied from 0.01 to 0.5, with a mean value of 0.246 (Alemu et al., 2020). AMOVA indicated that 73% of the variance was among individuals, 23% was between individuals, and 4% of the variation occurred among the 18 populations. This low genetic variation within individuals could be due to the fact that all of the accessions examined were from the same country. In addition, the low genetic variation could reflect the selection by farmers for desirable traits such as yield, stem strength, and resistance to biotic and abiotic stresses, and/or environmental adaptation and natural selection.

### 4.2 Linkage disequilibrium

The current analysis detected 33.6% of the total marker pairs with significant LD (*p* < 0.001) on both the A- and B-genomes, consistent with the 33%, 42%, and 43% reported by Rufo et al. (2019), Wang et al. (2019), and Roselló et al. (2019). The average squared allele correlation LD (r^2^) for the A-genome was slightly greater than that of B-genome (0.1802, and 0.1648), with a mean of 0.1723. Similarly, Fayaz et al. (2019) reported r^2^ values of 0.11, 0.11, and 0.12 for the whole genome, A-genome, and B-genome, respectively, in a collection of Iranian durum wheat landraces using 1,500 DArT markers. The lower average r^2^ of the B-genome compared with the A-genome is in accordance with the mean r^2^ of 0.12 for the A-genome and 0.11 for the B-genome reported by Roselló et al. (2019) in a Mediterranean collection of durum wheat. The average r^2^ values in the current study are lower than those reported by Roncallo et al. (2021) for a collection of Argentine durum wheat landraces (0.345, 0.278, and 0.302 for the A-genome, the B-genome and the whole genome, respectively). The LD decay of 14.6 Mb detected in this collection is similar to the values obtained with other durum wheat panels [11.8 Mb (Roncallo et al., 2021), 9.6 Mb (Wang et al., 2019), and 9.96 Mb (Taranto et al., 2020)]. However, it is lower than the LD of decay of 29.5 Mb reported in a collection of Argentine durum wheat landraces (Roncallo et al., 2021).

LD decay patterns varied between homoeologous chromosomes in genomes A and B. This result is consistent with that of Mengistu et al. (2016). These differences in LD decay patterns can be attributed to the different number of markers per homoeologous chromosomes. Mengistu et al. (2016) attributed such differences in LD decay to the different evolutionary forces in homoeologous pairs either by selection for QTL or genetic drift. A rapid decay such as reported in this study, is an indicator of a good genetic diversity of the panel studied, as well as the capacity to identify markers associated with traits (Mengistu et al., 2016).

LD half decay at fixed r^2^ of all chromosomes ranged from 2.9 to 7.3 Mb, indicating that the different genomic regions were subject to different selections and that the panel used in this study is diverse. The average LD half decay at fixed r^2^ of 5 Mb can be used as an up and down stream range to identify possible QTLs/genes. All MTAs within the latter range can be considered as one QTL, and that genes identified within the flanking region of the identified QTL can be considered as candidate genes.

The LD decay reported in our study is in accordance with other studies (Wu et al., 2021; Hao et al., 2022). Given the fact that LD decay varied between different chromosomes, and that different studies implement different methodologies for the LD estimation as well as different thresholds, the up- and downstream estimates remain approximate and should be treated with caution. As such, all identified candidate genes should be validated.

### 4.3 Clusters of Tunisian durum wheat accessions

Interestingly, the results indicated that of the 17 Mahmoudi accessions, 13 were commonly found in NJ cluster 5, UPGMA cluster 2, and Bayesian group 2, with the remaining four accessions distributed among other clusters or groups. Likewise, seven of the 12 Medea accessions were usually found in NJ cluster 2, UPGMA cluster 5, and Bayesian group 1, with the remaining five accessions distributed among other clusters or groups. This suggests that the four Mahmoudi and five Medea accessions distributed among other clusters or groups are either genetically distinct from the rest of the accessions in their corresponding populations, or that farmers and/or seed collectors mislabeled them. These results are consistent with those of Robbana et al. (2019), who concluded that of the six populations they tested, some of the Mahmoudi, Biskri, and Jenah Khetifah accessions were misclassified. Most of the Biskri (67%) and Mahmoudi (82%) accessions in the current study were grouped together in cluster 5, also consistent with the findings of Robbana et al. (2019) and Slim et al. (2019), who reported that the latter two landraces clustered together and constituted the same gene pool. The closeness of the Mahmoudi and Biskri populations was first reported by Boeuf (1932) based on glume and spike color. Miège (1950) suggested that this could be due to the exchange of these landraces with farmers from neighboring Algeria. The admixture group included at least one accession from each of the 18 populations, with the exception of Azizi, and some of these accessions occurred in different clusters. This could be due to mislabeling of the landraces during seed collection, the possible initial mixture of the landraces, or a mixture during threshing (Jaradat, 2013; Sahri et al., 2014; Robbana et al., 2019).

### 4.4 Phenotypic diversity

A moderate broad sense heritability (*h* ^*2*^) estimate for tan spot disease for the two cropping seasons where infected wheat stubble was added as an inoculum source was equal to 0.55. This suggested that 55% of the variability in tan spot disease in the tested accessions was due to genetic differences, while only 45% could be attributed to an environmental variance. This can be explained by the differences in disease pressure between the two trials. The *h* ^*2*^ estimate for tan spot disease for the three trials amounted to 0.28. This could be attributed to the different inoculation methods (natural and artificial with infested wheat stubble) between the trials as well as environmental effects leading to different levels of disease. These results are comparable to previous studies on tan spot adult plant resistance (Juliana et al., 2017; Muqaddasi et al., 2021). Indeed, Juliana et al. (2017) reported *h* ^*2*^ of 0.57 in a collection of 646 lines, while Muqaddasi et al. (2021) reported *h* ^*2*^ of 0.33 in a panel of 372 European lines.

Phenological traits data was not collected for all three trials; therefore, it was not included in our analyses. Although these traits were not included in our study, they may have had an effect on disease severity. While some previous studies on tan spot resistance have concluded that there is no association with phenological traits such as height and days to heading (Elias et al., 1989; Li et al., 2011; Kokhmetova et al., 2021; Laribi et al., 2021; Muqaddasi et al., 2021), others have reported a correlation between phenological traits and tan spot resistance/susceptibility (Fernandez et al., 2002; Kollers et al., 2014; Laribi et al., 2021; 2022b). The contradictory outcomes of these studies can be attributed to the use of different genetic material, the panel size, the different environments where the experiments were conducted, epidemiological factors, and the inoculation methods implemented. Therefore, the inclusion of such data could enable a better understanding of the contribution of phenological traits in tan spot severity.

The eight tan spot-resistant landraces identified in this study could serve as sources of resistance for elite durum wheat cultivars. Farmers prefer the Mahmoudi landrace in particular for its straw and grain yield (Ouaja et al., 2021). It has the ability to produce a high yield under the drought, salinity and heat stress conditions prevalent in southern Tunisia (Chamekh et al., 2015). Moreover, Mahmoudi has good technological and nutritional qualities (Lamine et al., 2022), it is a good source of favorable glutenin subunits (Ayed et al., 2010), and is among the most phenolic-rich landraces (Boukid et al., 2019).

### 4.5 Identification of co-localized markers on the A genome associated with tan spot resistance from previous studies

This study allowed the identification of several SNP markers associated with tan spot resistance.

Three SNP markers associated with tan spot were identified on chromosome 5A; ID 1109903, and ID 1135724. These three markers are different from the race-non specific QTL *QTs.fcu-5AL* reported on the same chromosome (138.4–140.1 cM) by Chu et al. (2008). However, marker ID 1135724 is closely positioned to two other QTLs (*QTs.fcu-5A.1* and *QTs.fcu-5A.2*) identified by Chu et al. (2010) on chromosome 5A and conferring resistance to races 1 and 2 of *P. tritici-repentis*. Marker ID 1109903 identified in this study on chromosome 5A at 81.5 cM falls within the range of *MQTL-5A.1* (81.1–95.9 cM) reported by Phuke et al. (2020); these markers explain 10.5% and 13.0% of phenotypic variance in resistance to tan spot, suggesting that they are similar or the same.

Markers ID 1078005, and ID 1092576 on chromosome 6A are located close to marker ID 3949961 conferring resistance to *P. tritici-repentis* race 1 (Dinglasan et al., 2021) at the adult stage under greenhouse conditions. These markers are also closely positioned to the markers identified by Phuke et al. (2020) at 596,903,177 bp, and by Lozano-Ramírez et al. (2022) at 599,622,814 bp. Marker ID 1074139 was positioned on chromosome 6A at 74.3 cM, only 3 cM distant from marker ID 100027398 positioned at 77.3 cM conferring resistance to tan spot disease (Lozano-Ramírez et al., 2022). The tan spot resistance genes *Tsr4* and *TsrAri* were both identified on chromosome 3A (Tadesse et al., 2010; Faris et al., 2013), yet no SNP markers were identified on the latter chromosome in this study. Markers ID 1090716 and ID 2248753 were identified on chromosome 4A and are not closely positioned to any previously identified markers related to tan spot resistance.

### 4.6 Identification of co-localized markers on the B genome associated with tan spot resistance from previous studies

*Tsc2*, which confers sensitivity to Ptr ToxB, was mapped on chromosome 2B at a 2.7 cM proximal distance from the SSR marker *Xmag681*, which co-segregates with marker *XTC339813* positioned at 44.6 and 0.6 cM distal from marker *XBE517745* at 66.9 cM (Abeysekara et al., 2010; Virdi et al., 2016). This places *Tsc2* between 44.6 and 66.3 cM. Three of the four SNP markers identified on chromosome 2B could be positioned at 83.2–94.8 cM on the consensus map, and therefore these three markers are different from *Tsc2*. According to Singh et al. (2016), the *Tsr6* gene located at 2BS and conferring recessive resistance to tan spot should be identical to *Tsc2*, hence these markers are distinct from *Tsr6* as well. Marker ID 1106958 was positioned at 40.7 cM, only 3.9 cM from *Tsc2*, and explained 16.2% of variance. Marker ID 4991617 is positioned on chromosome 2B at 86.0 cM only 4 cM and 0.6 cM distant from the two markers conferring resistance to tan spot disease, *wPt-7200* and *wPt-0950*, respectively (Chu et al., 2008; Gurung et al., 2011; Li et al., 2011; Patel et al., 2013). Marker ID 4991617 is only 0.5 cM distant from a marker QTL located 86.5–89.8 cM identified by Kokhmetova et al. (2021) explaining 8.7%–11.9% variation at the adult plant stage. Therefore, all these markers can be considered as one for tan spot resistance. Marker ID 1279775 was closely positioned to gene TRITD2Bv1G254430 coding for a zinc-finger (BTB/POZ) domain. The latter marker is positioned at 94.8 cM on chromosome 2B, 4 cM away from Marker ID wPt-4301 at 99.8 cM conferring resistance to tan spot at the seedling stage (Patel et al., 2013).

The *Tsn1* gene identified on chromosome 5B confers sensitivity to Ptr ToxA. This gene was absent in the Chinese Spring reference genome, but mapped between *Xfcp1* (54,923,624–549,236,608 bp) and *xfcp394* (549,950,246–549,950,630 bp) of the Chinese Spring reference genome (Faris et al., 2010). Marker ID 2271039 was located on chromosome 5B at 589,677,807 bp and was the closest in position to *Tsn1*. Liu et al. (2020) identified a marker *MQTL-5B.1* on chromosome 5B at a genetic range of 74.5–85.5 cM, explaining 21.0% of the resistance to tan spot. Marker ID 2271039 identified in this study falls within the latter range and explains 12.7% of phenotypic variance. Liu et al. (2020) identified a second marker *MQTL-5B.2* at 123.8 cM with a genetic range of 120.4–125.6 cM and explaining 21.5% of the resistance to tan spot. In addition, Perez-Lara et al. (2017) reported a genomic region associated with Ptr ToxB that mapped at 123–124 cM on chromosome 5B. Marker ID 2262945 identified in this study falls within this range and explains 8.2% of phenotypic variance in resistance to tan spot.

Marker *Xgwm285* was located on chromosome 3B [273,054,304 bp (Chinese spring) and 58.9 cM (consensus map)] and is closely related to *tsr2* and *tsr5* (Faris et al., 2013). All markers identified on chromosome 3B in this study are distant from *tsr2* and *tsr5* and, therefore, are distinct. Galagedara et al. (2020) identified a genomic region on chromosome 3B that was associated with resistance to four races of *P. tritici-repentis* and suggested that it was the same as the dominant race-nonspecific resistance gene *Tsr7* reported by Faris et al. (2020). Faris et al. (2020) developed semi-thermal asymmetric reverse PCR (STARP) markers for *Tsr7* designated as *fcp735* (470,381,965–470,382,065 bp) and *fcp736* (470,381,965–470,382,065 bp), which do not coincide with any of the markers identified in this study. Dinglasan et al. (2021) identified marker ID 1130858 on chromosome 7B at 483,755,047 bp from a bread wheat collection, which conferred resistance to race 1 at the seedling stage. Marker ID 1099093 identified in our study on chromosome 7B is relatively close to ID 1130858 at 469,742,950 bp.

Faris and Friesen (2005) identified a race-nonspecific QTL *QTsfcu-1BS*, but it was not possible to compare it with the SNP markers identified in this study since the flanking markers *Xgdm33* and *Xgdm125* were not identified in the reference genome. Marker ID 1074450 on chromosome 1B at 178,720,992 bp is located close to two markers [*wPt6833* (139,071,276 bp) and *wPt1328* (139,071,276 bp)] identified by Singh et al. (2016) and conferring resistance to race 1. This marker also coincides with marker ID 6045377 located at 51.29 cM on chromosome 1B reported by Lozano-Ramírez et al. (2022).

Marker ID 2266481 was identified by Lozano-Ramírez et al. (2022) on chromosome 6B at 602,745,555 bp on the Chinese spring wheat reference genome, near to our marker ID 1074139 at 604,537,957 bp. Marker ID4989018 located on chromosome 6A and explain 14% of the phenotypic variation had the highest allele effect (0.51) followed by markers ID 1139857 on chromosome 6A and ID 1099093 on chromosome 7B both explaining 11.4% of the phenotypic variation with an effect of 0.41 and 0.38, respectively. The absolute value of the effect size of most of the remaining SNPs ranged from 0.22 to 0.29. Understanding the effect size of MTAs on disease resistance can provide valuable insights into tan spot resistance mechanisms, as well as in the development of functional markers.

### 4.7 Putative candidate genes

Markers ID 1127995, ID 3064632 and ID 4991617 identified on chromosomes 5B, 3B and 2B, were all identified within genes TRITD5Bv1G222790, TRITD3Bv1G021190, and TRITD2Bv1G243350 coding for F-box proteins that are known to be associated with responses to biotic stresses and can act at different wheat development stages (Zhang et al., 2019). These proteins play an important role in protein regulation and degradation, plant photoperiodic signaling and hormone signaling (Zhang et al., 2019).

The markers ID 1135724 and ID 2262945 overlapped with genes TraesCS5B02G293300 and TRITD5Bv1G234990, respectively, coding for Zinc finger proteins. These proteins are reported as major transcription factors in *P. tritici-repentis*, triggering specific signaling pathways based on their up or downregulation (Adhikari et al., 2009).

Markers ID 1109903 and ID 1082485 overlapped with TraesCS5A02G376500 and TRITD2Bv1G087540 coding for BHLH proteins reported to be involved in plant pathogen defense by blocking the activity of plant transcription factors or directly promoting plant gene expression (Andersen et al., 2018).

Other identified markers were positioned within genes coding for disease resistance-related proteins, including GDSL domain-containing protein and NAC domain protein reported to be associated with the response to biotic and abiotic stresses (Andersen et al., 2018; Aiello et al., 2020; Ni et al., 2020; Zitnick-Anderson et al., 2020; Shen et al., 2022; Vranic et al., 2022).

## 5 Conclusion

The tan spot-resistant durum wheat landraces identified in this study could be used to improve resistance to this disease in high yielding elite cultivars. In addition, the genomic regions and markers found to be significantly associated with tan spot resistance at the adult plant stage provide more information regarding the genetic control of resistance to this disease in durum wheat. While some of these markers coincided with previously published markers associated with tan spot resistance, others represent novel markers. These results, together with previous studies, highlight the significance of chromosomes 2B, 5B and 6A as genomic regions associated with tan spot disease. The SNP markers identified in this study can be converted into Kompetitive allele specific PCR (KASP) markers and used to assess tan spot resistance as well as for marker assisted selection. These findings contribute to the pool of tools available for the development of tan spot-resistant durum wheat.

## Data Availability

The genotyping data presented in this study are available from the CIMMYT Research Data and Software Repository Network at https://hdl.handle.net/11529/10548953.
