# Supplementary figures and images for: Genome-wide association analysis of tan spot disease resistance in durum wheat accessions from Tunisia
Source: Front Genet. 2023 Oct 25;14:1231027. doi: 10.3389/fgene.2023.1231027 (PMC10631785; doi:10.3389/fgene.2023.1231027)

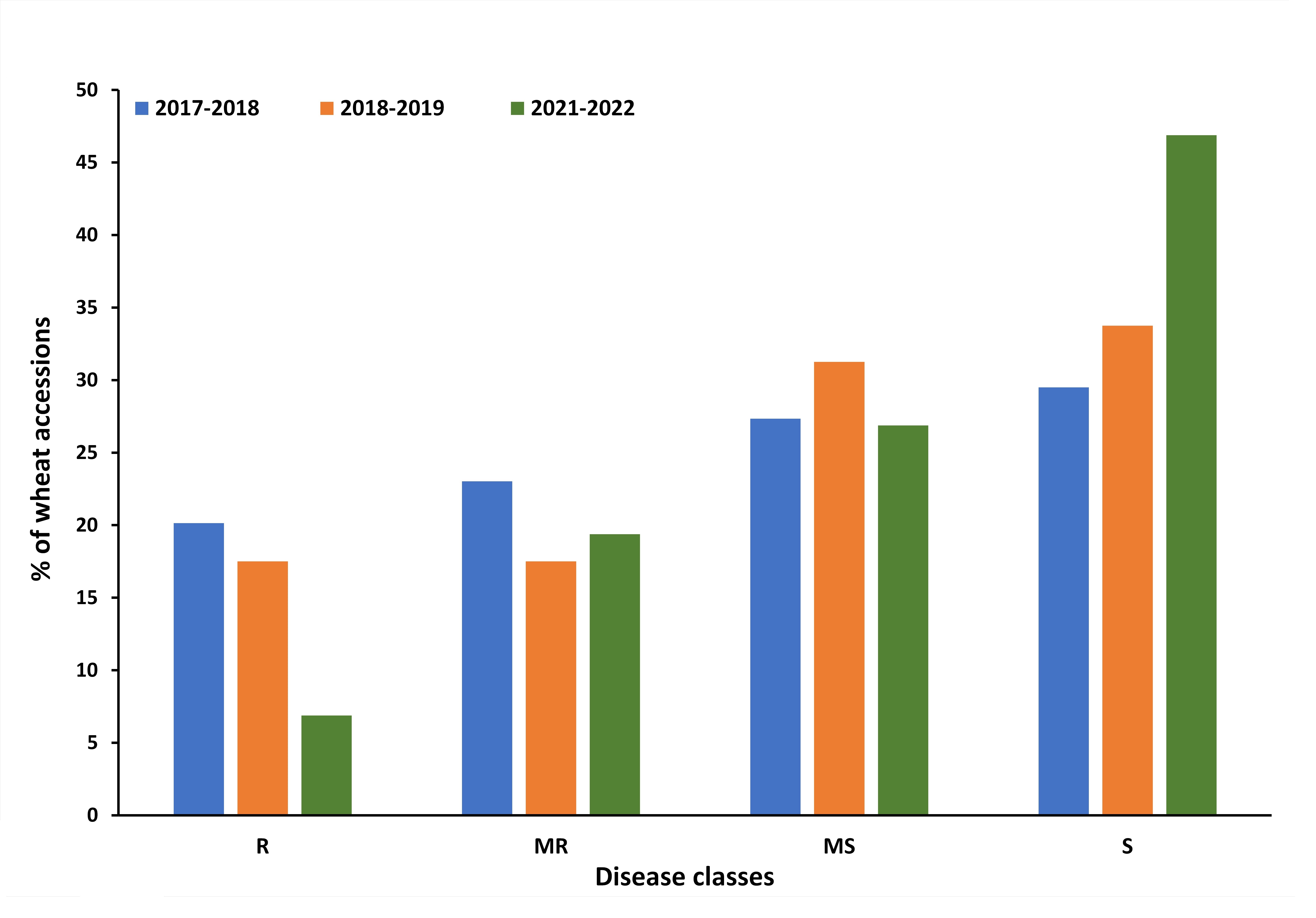

Supplement: Supplementary file 1 [file Image3.JPEG]

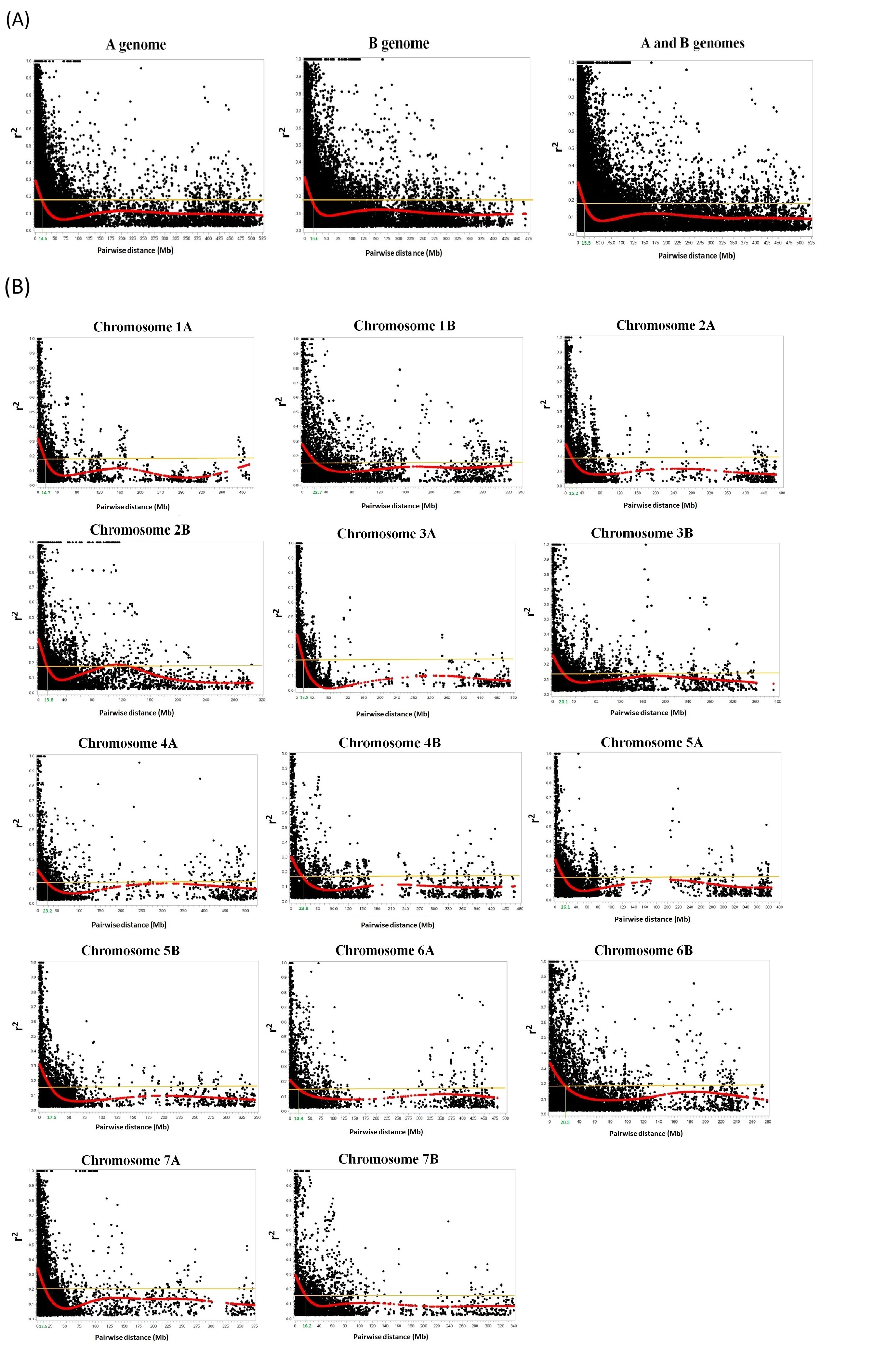

Supplement: Supplementary file 2 [file Image1.JPEG]
