# Supplementary material for: Genome-wide association analysis of tan spot disease resistance in durum wheat accessions from Tunisia
Source: Front Genet. 2023 Oct 25;14:1231027. doi: 10.3389/fgene.2023.1231027 (PMC10631785; doi:10.3389/fgene.2023.1231027)
Supplement: Supplementary file 4 [file DataSheet1.docx]

Supplementary Material

**Genome-wide association analyses of tan spot disease resistance in durum wheat accessions from Tunisia**

**Marwa Laribi^1,2†^, Rudolph Fredua-Agyeman^2†^, Sarrah Ben M’Barek^1,3^, Carolina P. Sansaloni^4^, Susanne Dreisigacker^4^, Fernanda M. Gamba^5^, Wided Abdedayem^1^, Meriem Nefzaoui^1^, Chayma Araar^1^, Sheau-Fang Hwang^2^, Amor H. Yahyaoui^1,6^* and Stephen E. Strelkov^2^***

*** Correspondence:**

Stephen Strelkov

[Strelkov@ualberta.ca](mailto:Strelkov@ualberta.ca)

Amor Hassine Yahyaoui

amor.yahyaoui@gmail.com

# Supplementary Figures and Tables

## Supplementary Figures

**Supplementary Figure 1.** Plots of correlation coefficient (r^2^) as a function of physical distance (in Mb) between pairs of SNP markers on chromosomes 1A, 1B, 2A, 2B, 3A, 3B, 4A, 4B, 5A, 5B, 6A, 6B, 7A, and 7B. Red curves represent the fit plots of the data points and the orange line represents the background linkage disequilibrium (BLD) or threshold line. The extent of LD decay was determined from projection of the intersection of the curves and the BLD line onto the physical distance.

**Supplementary Figure 2**. Distribution of allele frequency-based genetic diversity statistics (A), Polymorphic Information Content (PIC) (B), Minor Allele Frequency (MAF) (C), and Expected heterozygosity (He) or gene diversity (D) of 7654 SNP markers across 235 d urum wheat accessions from Tunisia based on their population’s assignments.

**Supplementary Figure 3.** QQ plots of all traits used in GWAS analysis with mixed linear model by using first three principal components as a fixed variate and kinship as a random variate. A–C: QQ plots of rAUDPC2017-2018, rAUDPC 2018–2019, and rAUDPC 2021–2022, respectively. D–G: QQ plots of average rAUDPC2017-2018 & 2018–2019, average rAUDPC2017-2018 & 2021–2022, average rAUDPC 2018–2019 & 2021–2022, and average for all three trials.

## Supplementary Tables

**Supplementary Table 1.** List of 235 durum wheat accessions from Tunisia included in this study of genetic diversity

| **Accession reference** | **USDA**  **Reference** | **Pedigree** | **Level of**  **improvement** | **Geographical origin^a^** | **Sub-population based on taxonomy^b^** | **Populations based on structure analysis^c^** | **Cluster NJ^d^** | **Cluster  UPGMA^e^** | **Kinship**  **Groups** | **Kinship**  **Sub-groups** |
| --- | --- | --- | --- | --- | --- | --- | --- | --- | --- | --- |
| 1 | CItr 3117 | Matmata | Landrace | Tunisia, Gabès | other-cvs | Group 1 | 1 | 5 | - | - |
| 2 | CItr 3137 | Adjini AC 1 | Landrace | Tunisia | Adjini | Group 1 | 3 | 1 | - | - |
| 3† | CItr 3138 | Adjini AP 1 | Landrace | Tunisia | Adjini | Group 1 | 3 | 1 | G1 | G1-2 |
| 4† | CItr 3139 | Adjini RC 1 | Landrace | Tunisia | Adjini | Group 1 | 1 | 5 | G1 | G1-2 |
| 5† | CItr 3140 | Agili Glabre AP 1 | Landrace | Tunisia | Agili | Admixture | 4 | 2 | G2 | G2-2 |
| 6† | CItr 3141 | Agili Glabre RC 1 | Landrace | Tunisia | Agili | Group 1 | 3 | 1 | G1 | G1-2 |
| 7† | CItr 3142 | Agili Glabre RC 2 | Landrace | Tunisia | Agili | Group 1 | 3 | 4 | G1 | G1-1 |
| 8† | CItr 3143 | Agili Glabre RP 1 | Landrace | Tunisia | Agili | Group 1 | 1 | 4 | G1 | G1-1 |
| 9† | CItr 3144 | Agili Glabre RP 2 | Landrace | Tunisia | Agili | Group 1 | 3 | 1 | G1 | G1-2 |
| 10† | CItr 3147 | Agili Pubescent AC 1 | Landrace | Tunisia | Agili | Group 1 | 1 | 4 | G1 | G1-1 |
| 11† | CItr 3148 | Agili Pubescent AC 2 | Landrace | Tunisia | Agili | Group 1 | 3 | 4 | G1 | G1-2 |
| 12† | CItr 3155 | Aouej AP 3 | Landrace | Tunisia | other-cvs | Group 2 | 5 | 2 | G2 | G2-1 |
| 13† | CItr 3156 | Azizi AC 2 | Landrace | Tunisia | Azizi | Group 2 | 5 | 2 | G2 | G2-1 |
| 14† | CItr 3158 | Azizi AP 1 | Landrace | Tunisia | Azizi | Group 1 | 2 | 5 | G1 | G1-1 |
| 15† | CItr 3160 | Azizi AP 3 | Landrace | Tunisia | Azizi | Group 2 | 5 | 2 | G2 | G2-1 |
| 16† | CItr 3162 | Azizi AP 5 | Landrace | Tunisia | Azizi | Group 1 | 2 | 5 | G1 | G1-1 |
| 17† | CItr 3163 | Azizi AP 6 | Landrace | Tunisia | Azizi | Group 1 | 2 | 5 | G1 | G1-1 |
| 18 | CItr 3166 | Azizi AP 9 | Landrace | Tunisia | Azizi | Group 1 | 2 | 5 | - | - |
| 19 | CItr 3169 | Baiada RP 1 | Landrace | Tunisia | other-cvs | Group 1 | 3 | 1 | - | - |
| 20† | CItr 3170 | Bidi AP 1 | Landrace | Tunisia | Bidi | Group 1 | 3 | 4 | G1 | G1-1 |
| 21† | CItr 3171 | Bidi AP 2 | Landrace | Tunisia | Bidi | Group 1 | 2 | 4 | G1 | G1-2 |
| 22† | CItr 3172 | Bidi AP 3 | Landrace | Tunisia | Bidi | Group 1 | 1 | 5 | G1 | G1-2 |
| 23† | CItr 3173 | Bidi AP 4 | Landrace | Tunisia | Bidi | Group 1 | 1 | 5 | G1 | G1-2 |
| 24† | CItr 3174 | Bidi AP 5 | Landrace | Tunisia | Bidi | Admixture | 3 | 1 | G1 | G1-2 |
| 25† | CItr 3175 | Biskri Glabre AC 2 | Landrace | Tunisia | Biskri | Group 1 | 2 | 5 | G1 | G1-2 |
| 26† | CItr 3176 | Biskri Glabre AP 1 | Landrace | Tunisia | Biskri | Group 2 | 5 | 2 | G1 | G1-2 |
| 27† | CItr 3177 | Biskri Glabre AP 2 | Landrace | Tunisia | Biskri | Admixture | 4 | 3 | G1 | G1-1 |
| 28† | CItr 3179 | Biskri Glabre RP 1 | Landrace | Tunisia | Biskri | Group 1 | 2 | 5 | G2 | G2-2 |
| 29† | CItr 3181 | Biskri Pubescent AC 1 | Landrace | Tunisia | Biskri | Group 2 | 5 | 2 | G1 | G1-1 |
| 30† | CItr 3183 | Biskri Pubescent AC 3 | Landrace | Tunisia | Biskri | Group 2 | 5 | 2 | G1 | G1-1 |
| 31† | CItr 3184 | Biskri Pubescent AP 1 | Landrace | Tunisia | Biskri | Group 2 | 5 | 2 | G2 | G2-2 |
| 32† | CItr 3185 | Biskri Velu RC 1 | Landrace | Tunisia | Biskri | Group 2 | 5 | 2 | G1 | G1-2 |
| 33† | CItr 3186 | Ble a Epi Cane RC 1 | Landrace | Tunisia | other-cvs | Admixture | 4 | 3 | G1 | G1-1 |
| 34 | CItr 3187 | Derberri AC 1 | Landrace | Tunisia | Derbessi | Group 1 | 1 | 4 | - | - |
| 35† | CItr 3189 | Derberri AC 3 | Landrace | Tunisia | Derbessi | Group 1 | 2 | 5 | G1 | G1-2 |
| 36 | CItr 3190 | Derberri AP 1 | Landrace | Tunisia | Derbessi | Group 1 | 1 | 4 | - | - |
| 37† | CItr 3192 | Derberri RC 1 | Landrace | Tunisia | Derbessi | Admixture | 4 | 3 | G1 | G1-2 |
| 38† | CItr 3193 | Hamira AC 1 | Landrace | Tunisia | Hamira | Group 1 | 3 | 4 | G1 | G1-1 |
| 39 | CItr 3195 | Hamira AC 3 | Landrace | Tunisia | Hamira | Group 1 | 1 | 4 | - | - |
| 40† | CItr 3196 | Hamira AC 4 | Landrace | Tunisia | Hamira | Group 1 | 3 | 1 | G1 | G1-1 |
| 41† | CItr 3197 | Hamira AC 5 | Landrace | Tunisia | Hamira | Group 1 | 3 | 1 | G1 | G1-1 |
| 42 | CItr 3198 | Jennah Rhetifa AP 2 | Landrace | Tunisia | Jenah_Khetifah | Group 1 | 1 | 4 | - | - |
| 43† | CItr 3199 | Jennah Rhetifa AP 4 | Landrace | Tunisia | Jenah_Khetifah | Group 1 | 1 | 4 | G1 | G1-2 |
| 44† | CItr 3200 | Jennah Rhetifa AP 9 | Landrace | Tunisia | Jenah_Khetifah | Group 1 | 1 | 4 | G2 | G2-1 |
| 45† | CItr 3201 | Jennah Rhetifa AP 10 | Landrace | Tunisia | Jenah_Khetifah | Group 1 | 1 | 4 | G2 | G2-1 |
| 46† | CItr 3202 | Jennah Rhetifa RP 1 | Landrace | Tunisia | Jenah_Khetifah | Admixture | 3 | 1 | G2 | G2-1 |
| 47† | CItr 3203 | Jennah Rhetifa RP 2 | Landrace | Tunisia | Jenah_Khetifah | Group 2 | 5 | 2 | G2 | G2-1 |
| 48† | CItr 3205 | Jennah Rhetifa RP 4 | Landrace | Tunisia | Jenah_Khetifah | Group 2 | 5 | 2 | G1 | G1-2 |
| 49† | CItr 3206 | Jennah Rhetifa RP 5 | Landrace | Tunisia | Jenah_Khetifah | Group 2 | 5 | 2 | G2 | G2-2 |
| 50 | CItr 3209 | Lebei Pubescent Bas AP 2 | Landrace | Tunisia | other-cvs | Group 2 | 5 | 2 | - | - |
| 51† | CItr 3210 | Lebei Pubescent AP 2 | Landrace | Tunisia | other-cvs | Group 1 | 1 | 5 | G2 | G2-1 |
| 52 | CItr 3211 | Lebei Velu Bas AC 1 | Landrace | Tunisia | other-cvs | Admixture | 4 | 3 | - | - |
| 53 | CItr 3212 | Lebei Velu Bas AC 2 | Landrace | Tunisia | other-cvs | Admixture | 4 | 3 | - | - |
| 54† | CItr 3221 | Louri AC 8 | Landrace | Tunisia | Souri | Group 1 | 3 | 1 | G2 | G2-2 |
| 55† | CItr 3222 | Louri AC 9 | Landrace | Tunisia | Souri | Admixture | 4 | 2 | G1 | G1-2 |
| 56† | CItr 3223 | Louri AP 2 | Landrace | Tunisia | Souri | Group 1 | 3 | 4 | G1 | G1-2 |
| 57† | CItr 3224 | Louri AP 3 | Landrace | Tunisia | Souri | Admixture | 3 | 1 | G1 | G1-2 |
| 58† | CItr 3225 | Louri AP 5 | Landrace | Tunisia | Souri | Admixture | 3 | 1 | G2 | G2-2 |
| 59† | CItr 3226 | Louri AP 6 | Landrace | Tunisia | Souri | Group 2 | 4 | 2 | G2 | G2-1 |
| 60 | CItr 3229 | Louri RP 3 | Landrace | Tunisia | Souri | Group 2 | 5 | 2 | - | - |
| 61 | CItr 3230 | Louri RP 4 | Landrace | Tunisia | Souri | Admixture | 4 | 3 | - | - |
| 62† | CItr 3231 | Louri RP 5 | Landrace | Tunisia | Souri | Admixture | 4 | 3 | G2 | G2-2 |
| 63† | CItr 3233 | Mahmoudi Glabre AC 1 | Landrace | Tunisia | Mahmoudi | Group 1 | 3 | 1 | G1 | G1-2 |
| 64† | CItr 3234 | Mahmoudi Glabre AC 4 | Landrace | Tunisia | Mahmoudi | Group 2 | 5 | 2 | G1 | G1-2 |
| 65 | CItr 3235 | Mahmoudi Glabre AP 1 | Landrace | Tunisia | Mahmoudi | Group 2 | 5 | 2 | - | - |
| 66† | CItr 3236 | Mahmoudi Glabre AP 2 | Landrace | Tunisia | Mahmoudi | Group 2 | 5 | 2 | G2 | G2-1 |
| 67† | CItr 3237 | Mahmoudi Glabre AP 3 | Landrace | Tunisia | Mahmoudi | Group 2 | 5 | 2 | G2 | G2-1 |
| 68† | CItr 3241 | Mahmoudi Glabre RP 2 | Landrace | Tunisia | Mahmoudi | Group 2 | 5 | 2 | G2 | G2-1 |
| 69† | CItr 3242 | Mahmoudi Pubescent AP 1 | Landrace | Tunisia | Mahmoudi | Group 1 | 1 | 5 | G2 | G2-1 |
| 70† | CItr 3243 | Medea AC 1 | Landrace | Tunisia | Medea | Group 1 | 2 | 5 | G1 | G1-2 |
| 71† | CItr 3244 | Medea AC 2 | Landrace | Tunisia | Medea | Group 1 | 1 | 4 | G1 | G1-1 |
| 72 | CItr 3245 | Medea AC 3 | Landrace | Tunisia | Medea | Group 2 | 5 | 2 | - | - |
| 73† | CItr 3246 | Medea AC 4 | Landrace | Tunisia | Medea | Group 1 | 2 | 5 | G1 | G1-1 |
| 74† | CItr 3248 | Medea AP 1 | Landrace | Tunisia | Medea | Group 1 | 2 | 5 | G1 | G1-2 |
| 75 | CItr 3249 | Medea AP 2 | Landrace | Tunisia | Medea | Group 1 | 2 | 5 | - | - |
| 76† | CItr 3251 | Medea AP 4 | Landrace | Tunisia | Medea | Admixture | 4 | 3 | G1 | G1-2 |
| 77 | CItr 3252 | Medea AP 6 | Landrace | Tunisia | Medea | Group 1 | 2 | 5 | - | - |
| 78† | CItr 3255 | Medea AP 9 | Landrace | Tunisia | Medea | Group 1 | 2 | 5 | G1 | G1-2 |
| 79† | CItr 3256 | Medea AP 10 | Landrace | Tunisia | Medea | Group 1 | 2 | 5 | G2 | G2-2 |
| 80† | CItr 3258 | Medea RP 1 | Landrace | Tunisia | Medea | Group 2 | 5 | 2 | G1 | G1-1 |
| 81† | CItr 3260 | Real Sorte AC 2 | Landrace | Tunisia | other-cvs | Group 1 | 2 | 5 | G1 | G1-2 |
| 82† | CItr 3261 | Real Sorte AC 3 | Landrace | Tunisia | other-cvs | Group 2 | 5 | 2 | G1 | G1-2 |
| 83 | CItr 3262 | Taganrog AC 1 | Landrace | Tunisia | other-cvs | Group 1 | 2 | 5 | - | - |
| 84 | CItr 3798 | Agini | Landrace | Tunisia, Beja | Agili | Group 1 | 2 | 5 | - | - |
| 85† | CItr 3809 | Mahmoudi | Landrace | Tunisia, Bizerte | Mahmoudi | Group 2 | 5 | 2 | G2 | G2-1 |
| 86† | CItr 3811 | Bidi | Landrace | Tunisia, Beja | Bidi | Group 2 | 5 | 2 | G1 | G1-1 |
| 87† | CItr 3816 | Mahmoudi | Landrace | Tunisia, Nabeul | Mahmoudi | Group 2 | 5 | 2 | G2 | G2-1 |
| 88† | CItr 3824 | Mahmoudi | Landrace | Tunisia, L'Ariana | Mahmoudi | Group 2 | 5 | 2 | G1 | G1-1 |
| 89† | CItr 3832 | Khetifa | Landrace | Tunisia | Jenah_Khetifah | Group 1 | 2 | 5 | G2 | G2-1 |
| 90 | CItr 3983 | ICM 313 | Landrace | Tunisia | other-cvs | Group 1 | 2 | 5 | - | - |
| 91† | CItr 3984 | ICM 314 | Landrace | Tunisia, Tataouine | other-cvs | Group 1 | 1 | 5 | G2 | G2-1 |
| 92† | CItr 6870 | Souri | Landrace | Tunisia | Souri | Group 1 | 2 | 5 | G2 | G2-1 |
| 93 | CItr 6875 | Sbei | Landrace | Tunisia | Sbei | Group 1 | 2 | 5 |  |  |
| 94† | CItr 6880 | Morocco | Landrace | Tunisia | other-cvs | Group 1 | 3 | 4 | G2 | G2-1 |
| 95† | CItr 15404 | Frigui | Landrace | Tunisia, Siliana | Frigui | Group 1 | 5 | 5 | G1 | G1-1 |
| 96† | CItr 15410 | 3124-10 | Landrace | Tunisia, Siliana | Unassigned Genotypes | Group 1 | 2 | 5 | G1 | G1-2 |
| 97 | CItr 15411 | 3320-18 | Landrace | Tunisia, Siliana | Unassigned Genotypes | Group 1 | 1 | 5 |  |  |
| 98† | CItr 15413 | Frigui | Landrace | Tunisia, Bizerte | Frigui | Group 1 | 1 | 5 | G1 | G1-1 |
| 99† | CItr 15415 | Ajili | Landrace | Tunisia, Bizerte | Agili | Group 1 | 1 | 4 | G1 | G1-1 |
| 100 | CItr 15418 | Hmira | Landrace | Tunisia, Kebili | Hamira | Admixture | 3 | 3 | - | - |
| 101 | CItr 15419 | Hmira | Landrace | Tunisia, Kebili | Hamira | Group 1 | 5 | 5 | - | - |
| 102 | CItr 15420 | Hmira | Landrace | Tunisia, Kebili | Hamira | Group 1 | 1 | 4 | - | - |
| 103 | CItr 15421 | Hmira | Landrace | Tunisia, Kebili | Hamira | Group 1 | 3 | 1 | - | - |
| 104 | CItr 15426 | Hmira | Landrace | Tunisia, Kebili | Hamira | Group 1 | 1 | 5 | - | - |
| 105† | CItr 15431 | 1346-27 | Landrace | Tunisia, Kebili | Unassigned Genotypes | Group 1 | 1 | 5 | G1 | G1-1 |
| 106† | CItr 15432 | Ajili | Landrace | Tunisia, Kebili | Agili | Group 2 | 4 | 2 | G1 | G1-2 |
| 107 | CItr 15436 | Hmira | Landrace | Tunisia, Kebili | Hamira | Group 1 | 3 | 4 | - | - |
| 108 | CItr 15439 | Djebali | Landrace | Tunisia, Kebili | other-cvs | Admixture | 3 | 1 | - | - |
| 109 | CItr 15442 | Hmira | Landrace | Tunisia, Kebili | Hamira | Group 1 | 1 | 5 | - | - |
| 110 | CItr 15443 | Hmira | Landrace | Tunisia, Kebili | Hamira | Admixture | 3 | 1 | - | - |
| 111 | CItr 15444 | Hmira | Landrace | Tunisia, Kebili | Hamira | Admixture | 4 | 3 | - | - |
| 112† | CItr 15449 | 3356-74 | Landrace | Tunisia, Kebili | Unassigned Genotypes | Group 1 | 1 | 5 | G1 | G1-2 |
| 113 | CItr 15450 | 3320-72 | Landrace | Tunisia, Kebili | Unassigned Genotypes | Group 2 | 5 | 2 | - | - |
| 114† | CItr 15451 | 3102-76 | Landrace | Tunisia, Kebili | Unassigned Genotypes | Group 2 | 5 | 2 | G1 | G1-1 |
| 115† | CItr 15452 | 2222-78 | Landrace | Tunisia, Kebili | Unassigned Genotypes | Group 1 | 3 | 1 | G1 | G1-2 |
| 116† | CItr 15453 | 2260-84 | Landrace | Tunisia, Kebili | Unassigned Genotypes | Group 1 | 3 | 1 | G2 | G2-2 |
| 117 | CItr 15454 | 1144-86 | Landrace | Tunisia, Kebili | Unassigned Genotypes | Group 1 | 1 | 4 | - | - |
| 118† | CItr 15455 | 2238-82 | Landrace | Tunisia, Kebili | Unassigned Genotypes | Group 1 | 3 | 1 | G1 | G1-2 |
| 119 | CItr 15457 | Hmira | Landrace | Tunisia, Kebili | Hamira | Group 1 | 1 | 4 | - | - |
| 120† | CItr 15458 | Frigui | Landrace | Tunisia, Kebili | Frigui | Group 1 | 1 | 4 | G2 | G2-1 |
| 121† | CItr 15459 | Frigui | Landrace | Tunisia, Kebili | Frigui | Group 1 | 2 | 5 | G1 | G1-2 |
| 122† | CItr 15460 | Hmira | Landrace | Tunisia, Kebili | Hamira | Group 1 | 1 | 4 | G1 | G1-2 |
| 123† | CItr 15461 | Frigui | Landrace | Tunisia, Kebili | Frigui | Group 1 | 1 | 5 | G1 | G1-2 |
| 124† | CItr 15462 | Frigui | Landrace | Tunisia, Kebili | Frigui | Group 1 | 1 | 4 | G1 | G1-1 |
| 125 | CItr 15464 | Frigui | Landrace | Tunisia, Kebili | Frigui | Group 1 | 1 | 4 | - | - |
| 126† | CItr 15469 | Farine | Landrace | Tunisia, Kebili | other-cvs | Group 2 | 4 | 2 | G1 | G1-1 |
| 127 | CItr 15470 | Mexiquain | Uncertain improvement status | Tunisia, Kebili | other-cvs | Group 1 | 1 | 5 | - | - |
| 128† | CItr 15472 | Derbazi | Landrace | Tunisia, Kebili | Derbessi | Admixture | 5 | 2 | G1 | G1-1 |
| 129† | CItr 15473 | Ajili | Landrace | Tunisia, Kebili | Agili | Group 2 | 4 | 2 | G1 | G1-2 |
| 130 | CItr 15476 | Ajili | Landrace | Tunisia, Kebili | Agili | Group 2 | 4 | 2 | - | - |
| 131† | CItr 15477 | Arbi | Landrace | Tunisia, Kebili | Arbi | Group 1 | 1 | 5 | G1 | G1-1 |
| 132 | CItr 15479 | Farine Arbi | Landrace | Tunisia, Kebili | other-cvs | Admixture | 4 | 3 | v |  |
| 133† | CItr 15480 | Arbi | Landrace | Tunisia, Kebili | Arbi | Group 2 | 4 | 2 | G1 | G1-1 |
| 134† | CItr 15482 | Afili Romani | Landrace | Tunisia, Kebili | other-cvs | Admixture | 5 | 3 | G2 | G2-2 |
| 135† | CItr 15483 | Bedi | Landrace | Tunisia, Kebili | Bidi | Admixture | 4 | 2 | G2 | G2-1 |
| 136 | CItr 15484 | Ajili | Landrace | Tunisia, Kebili | Agili | Group 1 | 3 | 4 | - | - |
| 137 | CItr 15486 | Frigui | Landrace | Tunisia | Frigui | Group 2 | 4 | 2 | - | - |
| 138 | CItr 15487 | Ajili | Landrace | Tunisia | Agili | Group 1 | 2 | 5 | - | - |
| 139† | CItr 15488 | Ajili | Landrace | Tunisia | Agili | Group 1 | 3 | 1 | G2 | G2-2 |
| 140 | CItr 15490 | Arbi | Landrace | Tunisia, Bizerte | Arbi | Admixture | 4 | 3 | - | - |
| 141† | CItr 15491 | Arbi | Landrace | Tunisia, Bizerte | Arbi | Admixture | 4 | 2 | G1 | G1-2 |
| 142† | CItr 15493 | Oued Kebir | Landrace | Tunisia, Jendouba | other-cvs | Group 2 | 4 | 2 | G2 | G2-2 |
| 143† | CItr 15494 | Beskri | Landrace | Tunisia, Tozeur | Biskri | Admixture | 5 | 5 | G2 | G2-2 |
| 144† | CItr 15495 | Beskri | Landrace | Tunisia, Tozeur | Biskri | Group 1 | 5 | 5 | G1 | G1-2 |
| 145 | CItr 15496 | Dhil Bhal Senlikat | Landrace | Tunisia, Tozeur | other-cvs | Group 2 | 5 | 2 | - | - |
| 146† | CItr 15497 | 6336-8 | Landrace | Tunisia, Tozeur | Unassigned Genotypes | Group 2 | 5 | 2 | G2 | G2-2 |
| 147† | CItr 15499 | Beskri | Landrace | Tunisia, Tozeur | Biskri | Group 2 | 5 | 2 | G1 | G1-1 |
| 148† | CItr 15501 | Mahmoudi | Landrace | Tunisia, Tozeur | Mahmoudi | Group 2 | 5 | 2 | G1 | G1-2 |
| 149† | CItr 15505 | 2116-16 | Landrace | Tunisia, Tozeur | Unassigned Genotypes | Group 2 | 5 | 2 | G2 | G2-2 |
| 150 | CItr 15506 | Sinlikat | Landrace | Tunisia, Tozeur | other-cvs | Group 1 | 2 | 5 |  |  |
| 151† | CItr 15509 | Melange | Landrace | Tunisia, Tozeur | other-cvs | Group 2 | 5 | 2 | G2 | G2-2 |
| 152† | CItr 15510 | Bedi | Landrace | Tunisia, Tozeur | Bidi | Admixture | 4 | 3 | G2 | G2-2 |
| 153 | CItr 15512 | Agin Sinlika | Landrace | Tunisia, Tozeur | Adjini | Group 2 | 5 | 2 | - | - |
| 154† | CItr 15514 | Hmira Makaou | Landrace | Tunisia, Tozeur | Hamira | Group 2 | 5 | 2 | G2 | G2-2 |
| 155† | CItr 15515 | Bedi | Landrace | Tunisia, Tozeur | Bidi | Group 2 | 5 | 2 | G1 | G1-2 |
| 156† | CItr 15516 | Bedi | Landrace | Tunisia, Tozeur | Bidi | Admixture | 4 | 3 | G2 | G2-1 |
| 157† | CItr 15519 | Ward Bled | Landrace | Tunisia, Tozeur | other-cvs | Group 1 | 5 | 5 | G2 | G2-1 |
| 158† | CItr 15895 | Ajili | Landrace | Tunisia, Bizerte | Agili | Group 1 | 5 | 4 | G2 | G2-1 |
| 159† | CItr 15896 | Frigui | Landrace | Tunisia, Bizerte | Frigui | Group 1 | 2 | 5 | G2 | G2-1 |
| 160† | CItr 15897 | Frigui | Landrace | Tunisia, Bizerte | Frigui | Group 1 | 5 | 3 | G1 | G1-1 |
| 161 | CItr 15909 | 3380-34 | Landrace | Tunisia, Kebili | Unassigned Genotypes | Admixture | 4 | 2 | - | - |
| 162† | CItr 15913 | Arbi | Landrace | Tunisia, Kebili | Arbi | Group 1 | 2 | 5 | G2 | G2-1 |
| 163 | PI 7653 | Abd-el-Kader | Landrace | Tunisia | other-cvs | Group 1 | 1 | 5 | - | - |
| 164 | PI 41036 | Biskri Smooth | Uncertain improvement status | Tunisia | Biskri | Group 1 | 3 | 1 | - | - |
| 165 | PI 41037 | Namira | Landrace | Tunisia | Hamira | Group 1 | 3 | 1 | - | - |
| 166† | PI 41038 | Realforte | Landrace | Tunisia | other-cvs | Group 1 | 3 | 1 | G2 | G2-2 |
| 167 | PI 41040 | Sbei | Landrace | Tunisia | Sbei | Group 1 | 1 | 5 | - | - |
| 168 | PI 41041 | Agili Pubescent | Cultivar | Tunisia | Agili | Admixture | 3 | 4 | - | - |
| 169† | PI 41042 | Sbei Smooth | Landrace | Tunisia | Sbei | Group 2 | 5 | 2 | G2 | G2-1 |
| 170† | PI 41043 | Taganrog | Landrace | Tunisia | other-cvs | Group 2 | 5 | 2 | G2 | G2-1 |
| 171† | PI 41044 | Mekki | Landrace | Tunisia | other-cvs | Group 1 | 3 | 1 | G2 | G2-2 |
| 172† | PI 41045 | Mahmoudi | Landrace | Tunisia | Mahmoudi | Admixture | 3 | 1 | G1 | G1-2 |
| 173 | PI 41047 | Azizi | Uncertain improvement status | Tunisia | Azizi | Group 1 | 1 | 5 | - | - |
| 174 | PI 41048 | Adjini | Uncertain improvement status | Tunisia | Adjini | Group 1 | 1 | 4 | - | - |
| 175 | PI 41049 | Allemand | Cultivar | Tunisia | other-cvs | Group 1 | 1 | 4 | - | - |
| 176 | PI 41051 | Souri | Landrace | Tunisia | Souri | Group 1 | 3 | 4 | - | - |
| 177† | PI 55529 | Adjini | Landrace | Tunisia | Adjini | Admixture | 5 | 3 | G2 | G2-2 |
| 178† | PI 55530 | Agili | Landrace | Tunisia | Agili | Admixture | 4 | 3 | G1 | G1-2 |
| 179† | PI 55531 | Agiliblanc 1 | Landrace | Tunisia | other-cvs | Admixture | 3 | 1 | G1 | G1-2 |
| 180† | PI 55533 | Aouedj | Landrace | Tunisia | other-cvs | Group 1 | 5 | 5 | G1 | G1-1 |
| 181† | PI 55534 | Derbessi | Landrace | Tunisia | Derbessi | Group 2 | 5 | 2 | G1 | G1-2 |
| 182† | PI 55535 | Biskri | Landrace | Tunisia | Biskri | Group 2 | 5 | 2 | G1 | G1-2 |
| 183 | PI 55536 | Hamira | Landrace | Tunisia | Hamira | Admixture | 5 | 3 | - | - |
| 184† | PI 55537 | Jenah Rhetifah | Landrace | Tunisia | Jenah_Khetifah | Admixture | 5 | 3 | G2 | G2-1 |
| 185† | PI 55538 | Mahmoudi AC 3 | Landrace | Tunisia | Mahmoudi | Group 2 | 5 | 2 | G2 | G2-1 |
| 186† | PI 55539 | Mahmoudi AP 5 | Landrace | Tunisia | Mahmoudi | Group 2 | 5 | 2 | G1 | G1-2 |
| 187† | PI 55540 | Medea | Landrace | Tunisia | Medea | Group 2 | 5 | 2 | G2 | G2-2 |
| 188† | PI 55541 | Mekki | Landrace | Tunisia | other-cvs | Group 2 | 5 | 2 | G2 | G2-2 |
| 189† | PI 55542 | Mekki | Landrace | Tunisia | other-cvs | Group 2 | 5 | 2 | G1 | G1-2 |
| 190† | PI 55544 | Souri | Landrace | Tunisia | Souri | Group 2 | 5 | 2 | G2 | G2-2 |
| 191 | PI 55545 | Sbei | Landrace | Tunisia | Sbei | Group 2 | 5 | 2 | - | - |
| 192† | PI 55546 | Sbei | Landrace | Tunisia | Sbei | Group 2 | 5 | 2 | G2 | G2-1 |
| 193 | PI 94698 | 387 | Landrace | Tunisia | Unassigned Genotypes | Group 2 | 5 | 2 | - | - |
| 194† | PI 150380 | Mahmoudi | Landrace | Tunisia | Mahmoudi | Group 2 | 5 | 2 | G2 | G2-1 |
| 195† | PI 150381 | Mahmoudi Hybrid | Breeding material | Tunisia | Mahmoudi | Group 2 | 5 | 2 | G2 | G2-2 |
| 196† | PI 185195 | Sbei 7 | Cultivar | Tunisia | Sbei | Admixture | 4 | 3 | G2 | G2-2 |
| 197† | PI 185412 | Agili Blanc 168 | Uncertain improvement status | Tunisia | Agili | Admixture | 4 | 3 | G2 | G2-1 |
| 198† | PI 191508 | Biskri AC2 | Breeding material | Tunisia | Biskri | Group 1 | 3 | 1 | G2 | G2-1 |
| 199 | PI 191938 | Derbessi | Landrace | Tunisia | Derbessi | Group 2 | 5 | 2 | - | - |
| 200† | PI 192517 | Jenah Retifah 24 | Cultivar | Tunisia | Jenah_Khetifah | Group 1 | 3 | 1 | G2 | G2-1 |
| 201 | PI 306572 | Chili 931 | Cultivar | Tunisia | Chili | Group 2 | 5 | 2 | - | - |
| 202† | PI 306575 | D 117 | Breeding material | Tunisia | Unassigned Genotypes | Group 1 | 3 | 1 | G2 | G2-1 |
| 203† | PI 306576 | D 240-5-1p-3 | Breeding material | Tunisia | Unassigned Genotypes | Group 1 | 1 | 5 | G2 | G2-1 |
| 204 | PI 189778 | Chili | Cultivar | Tunisia | Chili | Group 1 | 2 | 5 | - | - |
| 205† | PI 306576 | D 240-5-1p-3 | Breeding material | Tunisia | Unassigned Genotypes | Group 1 | 2 | 5 | G2 | G2-1 |
| 206† | PI 306577 | D 56-27-A | Breeding material | Tunisia | Unassigned Genotypes | Group 1 | 1 | 5 | G2 | G2-1 |
| 207† | PI 306579 | D 56-62-G | Breeding material | Tunisia | Unassigned Genotypes | Group 1 | 1 | 4 | G2 | G2-1 |
| 208† | PI 324935 | BD 1548 | Breeding material | Tunis, Tunisia | other-cvs | Group 1 | 1 | 4 | G2 | G2-1 |
| 209† | PI 324938 | D 56-89-C | Breeding material | Tunis, Tunisia | Unassigned Genotypes | Group 1 | 1 | 4 | G2 | G2-2 |
| 210 | PI 433751 | Badri | Cultivar | Tunisia | other-cvs | Group 1 | 3 | 4 |  |  |
| 211† | CHECK | karim | Cultivar | Tunisia | other-cvs | Group 1 | 1 | 4 | G1 | G1-2 |
| 212 | PI 520393 | Tunisian Durum 1 | Breeding material | Tunisia | other-cvs | Group 2 | 5 | 2 | - | - |
| 213 | PI 534335 | Chili | Landrace | Tunisia | Chili | Group 2 | 5 | 2 | - | - |
| 214 | PI 534336 | Chili | Landrace | Tunisia | Chili | Group 2 | 4 | 2 | - | - |
| 215 | PI 534338 | Biskri | Landrace | Tunisia | Biskri | Group 2 | 5 | 2 | - | - |
| 216 | PI 534342 | Chili | Landrace | Tunisia | Chili | Admixture | 5 | 3 | - | - |
| 217† | PI 534344 | Bidri | Landrace | Tunisia | other-cvs | Group 2 | 5 | 2 | G1 | G1-2 |
| 218 | PI 534347 | Chili | Landrace | Tunisia | Chili | Group 2 | 4 | 2 |  |  |
| 219 | PI 534348 | Chili | Landrace | Tunisia | Chili | Admixture | 5 | 1 | - | - |
| 220† | PI 534353 | MG 18141 | Landrace | Tunisia | MG | Group 2 | 4 | 2 | G1 | G1-2 |
| 221† | PI 534354 | MG 18142 | Landrace | Tunisia | MG | Group 2 | 4 | 2 | G1 | G1-2 |
| 222 | PI 534356 | MG 18144 | Landrace | Tunisia | MG | Group 2 | 4 | 2 | - | - |
| 223† | PI 534357 | MG 18145 | Landrace | Tunisia | MG | Group 2 | 4 | 2 | G1 | G1-1 |
| 224† | PI 534358 | MG 18146 | Landrace | Tunisia | MG | Group 2 | 4 | 2 | G2 | G2-1 |
| 225† | PI 534359 | Chili | Landrace | Tunisia | Chili | Group 2 | 4 | 2 | G2 | G2-2 |
| 226† | PI 534360 | MG 18148 | Landrace | Tunisia | MG | Group 2 | 4 | 2 | G2 | G2-2 |
| 227† | PI 534361 | MG 18149 | Landrace | Tunisia | MG | Admixture | 4 | 2 | G2 | G2-2 |
| 228† | PI 534362 | MG 18150 | Landrace | Tunisia | MG | Group 1 | 2 | 5 | G2 | G2-2 |
| 229† | PI 534365 | MG 18153 | Landrace | Tunisia | MG | Group 1 | 2 | 5 | G2 | G2-2 |
| 230† | PI 534368 | Mamhoudi | Landrace | Tunisia | Mahmoudi | Admixture | 5 | 3 | G2 | G2-2 |
| 231† | PI 534373 | MG 18161 | Landrace | Tunisia | MG | Admixture | 5 | 3 | G2 | G2-2 |
| 232 | PI 534374 | MG 18162 | Landrace | Tunisia | MG | Admixture | 5 | 3 | - | - |
| 233 | PI 534375 | MG 18163 | Landrace | Tunisia | MG | Admixture | 5 | 5 | - | - |
| 234 | PI 534376 | MG 18165 | Landrace | Tunisia | MG | Group 1 | 2 | 5 | - | - |
| 235 | PI 534377 | MG 18166 | Landrace | Tunisia | MG | Group 1 | 2 | 5 | - | - |

† Accessions retained for Genome-Wide Association study;^a^ Geographical origin: country name followed by the name of the area if available; **^b^Subpopulation based on taxonomy: genotypes with the same name were assigned to the same subpopulation;** ^c^Populations based on structure analysis: based on *STRUCTURE* results genotypes were assigned to group 1, group 2, and admixture; ^d^NJ, Neighbor joining; ^e^UPGMA, unweighted pair group method with arithmetic mean

**Supplementary Table 2**. SNP marker density and extent of intra-chromosomal linkage disequilibrium in wheat accessions from Tunisia.

| **Linkage group or Chromosome** | **Total # of SNP markers** | **# Filtered SNP markers** | **Lentgh covered (kb)** | **Average inter-SNP marker distance (kb)** | **Pairwise comparisons of all linked SNP markers** | **Number (%) of SNP pairs in significant LD ^ϕ^** | **Average r^2^ value/ chromosome** | **Estimated LD decay (Mb) ^ψ^** | **LD half decay (r^2^ = 0.1) (Mb)** |
| --- | --- | --- | --- | --- | --- | --- | --- | --- | --- |
| Chr1A | 2135 | 289 | 582748.4 | 2016.4 | 13175 | 5175 (39.3) | 0.1783 | 14.7 | 5.8 |
| Chr2A | 2883 | 413 | 774176.2 | 1874.5 | 19375 | 7109 (36.7) | 0.1859 | 15.2 | 4.2 |
| Chr3A | 2478 | 341 | 746343.6 | 2188.7 | 15775 | 5472 (34.7) | 0.2159 | 15.8 | 6.0 |
| Chr4A | 2110 | 256 | 741088.4 | 2894.9 | 11525 | 3913 (34.0) | 0.1483 | 23.2 | 2.9 |
| Chr5A | 2495 | 363 | 665188.1 | 1832.5 | 16875 | 6342 (37.6) | 0.1504 | 16.1 | 5.0 |
| Chr6A | 2001 | 239 | 615630.3 | 2575.9 | 10675 | 4488 (42.0) | 0.1556 | 14.8 | 3.2 |
| Chr7A | 3306 | 535 | 734505.1 | 1372.9 | 25475 | 8736 (34.3) | 0.2028 | 12.5 | 5.2 |
| **A-genome** | 17408 | 2436 | 4859680.2 | 1994.9 | 120525 | 41235 (34.2) | 0.1802 | 14.6 | 4.6 |
| Chr1B | 2764 | 341 | 679103.0 | 1991.5 | 15775 | 5813 (36.8) | 0.1628 | 23.7 | 5.1 |
| Chr2B | 3645 | 539 | 799339.3 | 1483.0 | 25675 | 9715 (37.8) | 0.1799 | 13.8 | 6.0 |
| Chr3B | 3114 | 402 | 845779.0 | 2103.9 | 18825 | 6763 (35.9) | 0.1492 | 20.1 | 3.0 |
| Chr4B | 1690 | 219 | 673771.8 | 3076.6 | 9675 | 3524 (36.4) | 0.1652 | 23.8 | 7.3 |
| Chr5B | 2873 | 375 | 694105.9 | 1850.9 | 17475 | 5567 (31.9) | 0.1529 | 17.5 | 5.2 |
| Chr6B | 2765 | 381 | 702625.2 | 1844.2 | 17775 | 7032 (39.6) | 0.1803 | 20.5 | 7.4 |
| Chr7B | 2977 | 377 | 746549.4 | 1980.2 | 17575 | 5171 (29.4) | 0.1506 | 16.2 | 4.0 |
| **B-genome** | 19828 | 2634 | 5141273.6 | 1951.9 | 130425 | 43585 (33.4) | 0.1648 | 16.6 | 5.4 |
| **A + B genome** | 37236 | 5070 | 10000953.7 | 1972.6 | 252225 | 84820 (33.6) | 0.1723 | 15.5 | 5 |
| **Scaffold (UN)** | 1312 | 128 | 486618.2 | 3801.7 | 5125 | 743 (14.5) | 0.1088 | 30.0 | - |
| **Not located (NA)** | 29991 | 3993 | - | - | - | - | - | - | - |
| **Total** | 68539 | 9191 | - | - | - | - | - | - | - |

^ϕ^ The number and percentage of SNP pairs in significant LD were determined from Chi-squared tests at *p*-value < 0.001.

^ψ^ The extent of LD decay was estimated from the projection of the intersection between the fitted curve of the data points and the 95^th^ percentile of unlinked r^2^ threshold line (background LD) onto the physical distance axis.

**Supplementary Table 3.** Marker information and genetic diversity statistics (Mean ± SE) for groups based on structure

| **Population** | **N^1^** | ***Na*^2^** | ***Ne*^3^** | ***I*^4^** | ***Ho*^5^** | ***He*^6^** | ***uHe*^7^** | ***F*^8^** | ***%P*^9^** |
| --- | --- | --- | --- | --- | --- | --- | --- | --- | --- |
| **Group 1** | 117  ±0.000 | 2.960  ±0.002 | 1.810  ±0.004 | 0.711  ±0.002 | 0.097  ±0.002 | 0.423  ±0.001 | 0.424  ±0.001 | 0.751  ±0.005 | 100% |
| **Group 2** | 74  ±0.000 | 2.903  ±0.003 | 1.776  ±0.005 | 0.694  ±0.002 | 0.091  ±0.002 | 0.408  ±0.002 | 0.411  ±0.002 | 0.770  ±0.005 | 100% |
| **Admixture** | 44  ±0.000 | 2.688  ±0.005 | 1.696  ±0.004 | 0.636  ±0.002 | 0.099  ±0.002 | 0.380  ±0.002 | 0.385  ±0.002 | 0.732  ±0.005 | 99.92% |
| **Total** | 78.333  ±0.198 | 2.850  ±0.002 | 1.761  ±0.003 | 0.680  ±0.001 | 0.096  ±0.001 | 0.404  ±0.001 | 0.407  ±0.001 | 0.751  ±0.003 | 99.97%  ±0.03% |

^1^N, Number of genotypes; ^2^*Na*, Number of Different Alleles; ^3^*Ne*, Number of Effective Alleles; ^4^*I*, Shannon's Information Index, ^5^*Ho*, Observed Heterozygosity; ^6^*He*, Expected Heterozygosity; ^7^*uHe*, Unbiased Expected Heterozygosity; ^8^*F*, Fixation Index; ^9^*%P*, percentage of polymorphic loci

**Supplementary Table 4.** Marker information and genetic diversity statistics (Mean ± SE) for 235 durum wheat accessions from Tunisia

| **Population** | **N^1^** | **Na^2^** | **Ne^3^** | **I^4^** | ***Ho*^5^** | ***He*^6^** | ***uHe*^7^** | **F^8^** | ***%P*^9^** |
| --- | --- | --- | --- | --- | --- | --- | --- | --- | --- |
| **Adjini** | 6 | 1.992  ±0.006 | 1.617  ±0.005 | 0.518  ±0.003 | 0.122  ±0.002 | 0.337  ±0.002 | 0.367  ±0.002 | 0.610  ±0.006 | 85.80% |
| **Agili** | 19 | 2.425  ±0.006 | 1.734  ±0.005 | 0.627  ±0.003 | 0.096  ±0.002 | 0.389  ±0.002 | 0.399  ±0.002 | 0.726  ±0.005 | 98.21% |
| **Arbi** | 5 | 1.855  ±0.007 | 1.551  ±0.005 | 0.457  ±0.004 | 0.095  ±0.002 | 0.297  ±0.002 | 0.330  ±0.003 | 0.661  ±0.007 | 73.99% |
| **Azizi** | 7 | 2.015  ±0.007 | 1.619  ±0.005 | 0.516  ±0.003 | 0.105  ±0.002 | 0.331  ±0.002 | 0.357  ±0.002 | 0.655  ±0.006 | 83.34% |
| **Bidi** | 10 | 2.197  ±0.007 | 1.699  ±0.005 | 0.575  ±0.002 | 0.101  ±0.002 | 0.362  ±0.002 | 0.381  ±0.002 | 0.692  ±0.006 | 89.89% |
| **Biskri** | 15 | 2.745  ±0.005 | 1.805  ±0.005 | 0.693  ±0.003 | 0.090  ±0.002 | 0.408  ±0.002 | 0.422  ±0.002 | 0.784  ±0.005 | 98.94% |
| **Chili** | 8 | 2.349  ±0.007 | 1.656  ±0.005 | 0.592  ±0.003 | 0.081  ±0.002 | 0.358  ±0.002 | 0.382  ±0.002 | 0.786  ±0.005 | 93.09% |
| **Derbessi** | 7 | 2.172  ±0.007 | 1.748  ±0.006 | 0.594  ±0.003 | 0.097  ±0.002 | 0.378  ±0.002 | 0.408  ±0.002 | 0.709  ±0.006 | 89.56% |
| **Frigui** | 10 | 2.090  ±0.007 | 1.621  ±0.005 | 0.518  ±0.003 | 0.090  ±0.002 | 0.328  ±0.002 | 0.345  ±0.002 | 0.704  ±0.006 | 85.03% |
| **Hamira** | 18 | 2.491  ±0.006 | 1.797  ±0.005 | 0.659  ±0.003 | 0.104  ±0.002 | 0.407  ±0.002 | 0.419  ±0.002 | 0.721  ±0.005 | 98.82% |
| **Jenah Khetifah** | 11 | 2.226  ±0.007 | 1.704  ±0.006 | 0.585  ±0.003 | 0.094  ±0.003 | 0.368  ±0.002 | 0.385  ±0.002 | 0.721  ±0.006 | 90.91% |
| **Mahmoudi** | 17 | 2.774  ±0.05 | 1.943  ±0.005 | 0.756 ±0.003 | 0.084  ±0.002 | 0.452  ±0.002 | 0.466  ±0.002 | 0.804  ±0.004 | 99.39% |
| **Medea** | 12 | 2.310  ±0.006 | 1.689  ±0.005 | 0.592  ±0.003 | 0.092  ±0.002 | 0.366  ±0.002 | 0.382  ±0.002 | 0.732  ±0.006 | 94.54% |
| **MG** | 14 | 2.430  ±0.007 | 1.649  ±0.005 | 0.584  ±0.003 | 0.084  ±0.002 | 0.350  ±0.002 | 0.363  ±0.002 | 0.762  ±0.005 | 94.19% |
| **Other-cvs** | 38 | 2.606  ±0.006 | 1.657  ±0.004 | 0.607  ±0.002 | 0.095  ±0.002 | 0.363  ±0.002 | 0.368  ±0.002 | 0.734  ±0.005 | 99.70% |
| **Sbei** | 6 | 2.068  ±0.007 | 1.678  ±0.00 | 0.553  ±0.003 | 0.101  ±0.002 | 0.355  ±0.002 | 0.387  ±0.002 | 0.687  ±0.006 | 86.73% |
| **Souri** | 12 | 2.463  ±0.006 | 1.806  ±0.005 | 0.666  ±0.003 | 0.102  ±0.002 | 0.411  ±0.002 | 0.428  ±0.002 | 0.726  ±0.005 | 97.86% |
| **Unassigned Genotypes** | 20 | 2.314  ±0.006 | 1.598  ±0.005 | 0.546  ±0.003 | 0.100  ±0.002 | 0.336  ±0.002 | 0.344  ±0.002 | 0.702  ±0.006 | 95.21% |
| **Total** | 235 | 1.991  ±0.001 | 1.619  ±0.001 | 0.519  ±0.001 | 0.113  ±0.001 | 0.337  ±0.001 | 0.368  ±0.001 | 0.637  ±0.001 | 91.95%  ±1.65% |

^1^N, Number of genotypes; ^2^*Na*, Number of Different Alleles; ^3^*Ne*, Number of Effective Alleles; ^4^*I*, Shannon's Information Index, ^5^*Ho*, Observed Heterozygosity; ^6^*He*, Expected Heterozygosity; ^7^*uHe*, Unbiased Expected Heterozygosity; ^8^*F*, Fixation Index; ^9^*%P*, percentage of polymorphic loci

**Supplementary Table 5.** Gene Diversity (D). Polymorphic information content (PIC). and Minor Allele Frequency (MAF) for assigned populations across 7654 SNP markers

| **Population** | **Gene Diversity (D)** | **Polymorphic information content (PIC)** | **Minor Allele Frequency (MAF)** |
| --- | --- | --- | --- |
| Adjini | 0.305 | 0.295 | 0.270 |
| Agili | 0.363 | 0.321 | 0.277 |
| Arbi | 0.357 | 0.370 | 0.335 |
| Azizi | 0.276 | 0.259 | 0.238 |
| Bidi | 0.379 | 0.355 | 0.309 |
| Biskri | 0.315 | 0.284 | 0.236 |
| Chili | 0.275 | 0.257 | 0.224 |
| Derbessi | 0.291 | 0.274 | 0.247 |
| Frigui | 0.365 | 0.338 | 0.300 |
| Hamira | 0.381 | 0.340 | 0.295 |
| Jenah_Khetifah | 0.340 | 0.310 | 0.274 |
| Mahmoudi | 0.266 | 0.246 | 0.185 |
| Medea | 0.368 | 0.335 | 0.299 |
| MG | 0.311 | 0.276 | 0.240 |
| Other-cvs | 0.406 | 0.359 | 0.297 |
| Sbei | 0.302 | 0.293 | 0.264 |
| Souri | 0.391 | 0.368 | 0.308 |
| Unassigned Genotypes | 0.395 | 0.352 | 0.303 |
| Average | 0.338 | 0.313 | 0.272 |

**Supplementary Table 6.** Pairwise correlation of the fixation index or F_ST_ values between populations of durum wheat accessions from Tunisia

|  | **Adjini** | **Agili** | **Arbi** | **Azizi** | **Bidi** | **Biskri** | **Chili** | **Derbessi** | **Frigui** | **Hamira** | **Jenah_**  **Khetifah** | **Mahmoudi** | **Medea** | **MG** | **Other-cvs** | **Sbei** | **Souri** | **Unassigned Geno** |
| --- | --- | --- | --- | --- | --- | --- | --- | --- | --- | --- | --- | --- | --- | --- | --- | --- | --- | --- |
| **Adjini** | 0.000 | 10.690 | 2.522 | 4.209 | 7.222 | 4.299 | 1.943 | 3.659 | 2.338 | 7.783 | 6.080 | 8.395 | 5.714 | 2.042 | 3.508 | _ | 9.257 | 2.306 |
| **Agili** | 0.023 | 0.000 | 8.604 | 12.223 | 7.213 | 5.811 | 3.365 | 4.813 | 14.948 | 6.312 | 6.432 | 7.271 | 6.664 | 3.202 | 5.451 | 39.161 | 17.854 | 2.937 |
| **Arbi** | 0.090 | 0.028 | 0.000 | 79.831 | 2.288 | - | 18.907 | 4.981 | - | 2.341 | 6.029 | 6.422 | - | - | - | 97.422 | 9.603 | 87.371 |
| **Azizi** | 0.056 | 0.020 | 0.003 | 0.000 | 7.546 | - | 13.967 | 3.253 | 6.261 | 3.972 | 9.431 | 86.946 | - | 15.000 | - | 74.701 | - | 7.123 |
| **Bidi** | 0.033 | 0.033 | 0.099 | 0.032 | 0.000 | 3.609 | 2.062 | 2.791 | 2.162 | 4.822 | 3.838 | 10.730 | 3.659 | 1.952 | 2.867 | 5.529 | 8.287 | 1.772 |
| **Biskri** | 0.055 | 0.041 | 0.000 | 0.000 | 0.065 | 0.000 | 16.218 | 3.461 | 5.322 | 2.859 | 10.395 | 10.251 | 84.734 | 18.157 | 27.786 | - | 14.101 | 6.686 |
| **Chili** | 0.114 | 0.069 | 0.013 | 0.018 | 0.108 | 0.015 | 0.000 | 2.477 | 15.124 | 1.947 | 2.969 | 3.258 | 4.737 | - | 14.232 | 7.798 | 5.736 | 2.546 |
| **Derbessi** | 0.064 | 0.049 | 0.048 | 0.071 | 0.082 | 0.067 | 0.092 | 0.000 | 3.881 | 6.814 | 3.225 | 4.422 | 3.867 | 2.783 | 2.755 | 5.599 | 6.471 | 2.471 |
| **Frigui** | 0.097 | 0.016 | 0.000 | 0.038 | 0.104 | 0.045 | 0.016 | 0.061 | 0.000 | 2.479 | 2.880 | 2.819 | 4.200 | 7.819 | 6.899 | 5.929 | 5.137 | 2.752 |
| **Hamira** | 0.031 | 0.038 | 0.097 | 0.059 | 0.049 | 0.080 | 0.114 | 0.035 | 0.092 | 0.000 | 4.782 | 5.964 | 3.415 | 1.917 | 2.566 | 5.541 | 7.236 | 1.889 |
| **Jenah_Khetifah** | 0.039 | 0.037 | 0.040 | 0.026 | 0.061 | 0.023 | 0.078 | 0.072 | 0.080 | 0.050 | 0.000 | 12.867 | 9.862 | 3.650 | 7.717 | 23.670 | 33.510 | 4.076 |
| **Mahmoudi** | 0.029 | 0.033 | 0.037 | 0.003 | 0.023 | 0.024 | 0.071 | 0.054 | 0.081 | 0.040 | 0.019 | 0.000 | 15.830 | 3.522 | 3.894 | 22.304 | 87.667 | 3.497 |
| **Medea** | 0.042 | 0.036 | 0.000 | 0.000 | 0.064 | 0.003 | 0.050 | 0.061 | 0.056 | 0.068 | 0.025 | 0.016 | 0.000 | 15.340 | 14.391 | - | 15.776 | 18.890 |
| **MG** | 0.109 | 0.072 | 0.000 | 0.016 | 0.114 | 0.014 | 0.000 | 0.082 | 0.031 | 0.115 | 0.064 | 0.066 | 0.016 | 0.000 | 38.129 | 6.902 | 5.638 | 5.619 |
| **Other-cvs** | 0.067 | 0.044 | 0.000 | 0.000 | 0.080 | 0.009 | 0.017 | 0.083 | 0.035 | 0.089 | 0.031 | 0.060 | 0.017 | 0.007 | 0.000 | 25.104 | 10.377 | 7.094 |
| **Sbei** | 0.000 | 0.006 | 0.003 | 0.003 | 0.043 | 0.000 | 0.031 | 0.043 | 0.040 | 0.043 | 0.010 | 0.011 | 0.000 | 0.035 | 0.010 | 0.000 | - | 8.447 |
| **Souri** | 0.026 | 0.014 | 0.025 | 0.000 | 0.029 | 0.017 | 0.042 | 0.037 | 0.046 | 0.033 | 0.007 | 0.003 | 0.016 | 0.042 | 0.024 | 0.000 | 0.000 | 5.061 |
| **Unassigned Genotypes** | 0.098 | 0.078 | 0.003 | 0.034 | 0.124 | 0.036 | 0.089 | 0.092 | 0.083 | 0.117 | 0.058 | 0.067 | 0.013 | 0.043 | 0.034 | 0.029 | 0.047 | 0.000 |

**Supplementary Table 7:** Pairwise unbiased genetic distance (Nei uD) comparison between the 18 populations defined based on their taxonomy

| **Population 1** | **Population 2** | **Nei uD** |
| --- | --- | --- |
| Arbi | MG | 0.013 |
| MG | other | 0.015 |
| Biskri | other | 0.016 |
| Azizi | Other cv | 0.018 |
| Arbi | Other cv | 0.020 |
| Medea | Unassigned Genotypes | 0.022 |
| Chili | MG | 0.022 |
| Medea | Other cv | 0.023 |
| Biskri | Medea | 0.023 |
| Azizi | Biskri | 0.026 |
| Arbi | Medea | 0.026 |
| other | Souri | 0.027 |
| Azizi | Medea | 0.027 |
| Arbi | Frigui | 0.027 |
| Biskri | MG | 0.027 |
| Mahmoudi | Souri | 0.027 |
| other | Sbei | 0.028 |
| Arbi | Unassigned Genotypes | 0.028 |
| Azizi | Souri | 0.028 |
| Biskri | Sbei | 0.028 |
| Chili | other | 0.028 |
| other | Unassigned Genotypes | 0.029 |
| Arbi | Biskri | 0.029 |
| Agili | Souri | 0.029 |
| Medea | MG | 0.029 |
| Agili | Frigui | 0.030 |
| Jenah_Khetifah | Souri | 0.030 |
| Medea | Sbei | 0.032 |
| Jenah_Khetifah | other | 0.033 |
| Mahmoudi | Medea | 0.034 |
| Medea | Souri | 0.035 |
| Agili | Sbei | 0.035 |
| Azizi | Mahmoudi | 0.035 |
| Adjini | Sbei | 0.035 |
| Azizi | MG | 0.036 |
| Biskri | Unassigned Genotypes | 0.036 |
| Frigui | other | 0.036 |
| Sbei | Souri | 0.036 |
| Chili | Frigui | 0.037 |
| Biskri | Souri | 0.037 |
| Agili | Other cv | 0.038 |
| MG | Unassigned Genotypes | 0.038 |
| Frigui | MG | 0.039 |
| Sbei | Unassigned Genotypes | 0.039 |
| Jenah_Khetifah | Mahmoudi | 0.039 |
| Azizi | Unassigned Genotypes | 0.039 |
| Agili | Azizi | 0.040 |
| Jenah_Khetifah | Medea | 0.040 |
| Biskri | Jenah_Khetifah | 0.040 |
| Biskri | Chili | 0.040 |
| Azizi | Sbei | 0.041 |
| Arbi | Azizi | 0.041 |
| Biskri | Mahmoudi | 0.042 |
| Jenah_Khetifah | Sbei | 0.042 |
| Agili | Mahmoudi | 0.044 |
| Agili | Medea | 0.044 |
| Bidi | Mahmoudi | 0.044 |
| Agili | Hamira | 0.044 |
| Agili | Bidi | 0.044 |
| Souri | Unassigned Genotypes | 0.045 |
| Adjini | Agili | 0.045 |
| Agili | Jenah_Khetifah | 0.046 |
| Azizi | Chili | 0.046 |
| Azizi | Jenah_Khetifah | 0.047 |
| Hamira | Souri | 0.048 |
| Bidi | Souri | 0.048 |
| Agili | Biskri | 0.048 |
| Arbi | Sbei | 0.049 |
| MG | Souri | 0.050 |
| Mahmoudi | Sbei | 0.050 |
| Azizi | Frigui | 0.050 |
| Jenah_Khetifah | Unassigned Genotypes | 0.051 |
| Azizi | Bidi | 0.051 |
| Arbi | Chili | 0.051 |
| MG | Sbei | 0.052 |
| Mahmoudi | Other cv | 0.053 |
| Hamira | Mahmoudi | 0.053 |
| Biskri | Frigui | 0.054 |
| Agili | Arbi | 0.054 |
| Adjini | Hamira | 0.055 |
| Adjini | Bidi | 0.056 |
| Frigui | Souri | 0.056 |
| Adjini | Souri | 0.057 |
| Frigui | Sbei | 0.057 |
| Derbessi | Hamira | 0.058 |
| Hamira | Jenah_Khetifah | 0.059 |
| Frigui | Medea | 0.059 |
| Bidi | Hamira | 0.059 |
| Arbi | Souri | 0.060 |
| Mahmoudi | Unassigned Genotypes | 0.060 |
| Adjini | Jenah_Khetifah | 0.061 |
| Adjini | Medea | 0.061 |
| Adjini | Mahmoudi | 0.063 |
| Chili | Medea | 0.064 |
| Jenah_Khetifah | MG | 0.064 |
| Chili | Sbei | 0.064 |
| Agili | Unassigned Genotypes | 0.064 |
| Arbi | Jenah_Khetifah | 0.064 |
| Chili | Souri | 0.064 |
| Agili | Derbessi | 0.065 |
| Adjini | Other cv | 0.066 |
| Frigui | Unassigned Genotypes | 0.066 |
| Bidi | Sbei | 0.067 |
| Derbessi | Souri | 0.067 |
| Hamira | Sbei | 0.067 |
| Agili | MG | 0.067 |
| Bidi | Other cv | 0.069 |
| Bidi | Jenah_Khetifah | 0.069 |
| Bidi | Medea | 0.070 |
| Derbessi | Frigui | 0.071 |
| Mahmoudi | MG | 0.072 |
| Hamira | Medea | 0.072 |
| Azizi | Hamira | 0.073 |
| Hamira | other | 0.074 |
| Adjini | Azizi | 0.074 |
| Bidi | Biskri | 0.075 |
| Arbi | Mahmoudi | 0.076 |
| Agili | Chili | 0.077 |
| Derbessi | Medea | 0.077 |
| Chili | Unassigned Genotypes | 0.077 |
| Derbessi | Other cv | 0.077 |
| Frigui | Jenah_Khetifah | 0.077 |
| Adjini | Biskri | 0.078 |
| Derbessi | Sbei | 0.080 |
| Arbi | Derbessi | 0.081 |
| Derbessi | Unassigned Genotypes | 0.082 |
| Adjini | Unassigned Genotypes | 0.085 |
| Derbessi | Mahmoudi | 0.087 |
| Biskri | Hamira | 0.087 |
| Derbessi | MG | 0.087 |
| Chili | Jenah_Khetifah | 0.088 |
| Derbessi | Jenah_Khetifah | 0.089 |
| Frigui | Hamira | 0.090 |
| Biskri | Derbessi | 0.090 |
| Frigui | Mahmoudi | 0.091 |
| Azizi | Derbessi | 0.092 |
| Adjini | Derbessi | 0.092 |
| Adjini | Frigui | 0.095 |
| Bidi | Frigui | 0.096 |
| Chili | Mahmoudi | 0.096 |
| Bidi | Derbessi | 0.098 |
| Hamira | Unassigned Genotypes | 0.098 |
| Bidi | Unassigned Genotypes | 0.101 |
| Bidi | MG | 0.104 |
| Adjini | Arbi | 0.104 |
| Adjini | MG | 0.107 |
| Arbi | Bidi | 0.108 |
| Hamira | MG | 0.108 |
| Arbi | Hamira | 0.114 |
| Bidi | Chili | 0.114 |
| Chili | Derbessi | 0.115 |
| Chili | Hamira | 0.124 |
| Adjini | Chili | 0.128 |

**Supplementary Table 8.** Candidate genes for significant marker-trait associations identified from the *Triticum turgidum* (Svevo. v1) and *Triticum aestivum* (IWGSC) reference genomes. Data were obtained from Emsembl (<https://plants.ensembl.org/>)

| **SNP marker** | **chromosome** | **Gene** | **Description** |
| --- | --- | --- | --- |
| 100050780 | 1B | TRITD1Bv1G128350 | UDP-Glycosyltransferase |
|  |  | TraesCS1A02G206600 |  |
| 5577017 | 3B | TRITD3Bv1G183390 | Fatty acid metabolism regulator protein G |
|  |  | TraesCS3B02G356300 | unknown |
|  |  |  |  |
|  |  |  |  |
| 1109903^†^ | 5A | TraesCS5A02G376500 | Myc-type, basic helix-loop-helix (bHLH) domain |
| 2276400 | 5A | TRITD5Av1G124440 | GDSL lipase/esterase |
|  |  | TraesCS5A02G167400 |  |
| 1127995 | 5B | TRITD5Bv1G222790 | F-box family protein |
|  |  | TraesCS5B02G669300LC | unknown |
| 1135724^†*^ | 5B | TraesCS5B02G293300 | Zinc finger |
| 2262945 | 5B | TRITD5Bv1G234990 | Plant regulator RWP-RK family protein, putative |
|  |  | TraesCS5B02G501500 | Protein NLP Zinc finger, BED-type |
| 2271039^†*^ | 5B | TRITD5Bv1G206810 | NAC domain protein |
|  |  | TraesCS5B02G415400 |  |
| 990930^†*^ | 6A | TraesCS6A02G049900 | Glutamate receptor |
| 1139857^†*^ | 6A | TRITD6Av1G009250 | Glutamate receptor |
| 1099093 | 7B | TraesCS7B02G254700 | unknown |
| 1279775 | 2B | TRITD2Bv1G254430 | BTB/POZ domain-containing protein |
| 1106958 | 2B | TRITD2Bv1G024670 | Protein trichome birefringence |
|  |  | TraesCS2B02G093500 |  |
| 4991617 | 2B | TRITD2Bv1G243350 | unknown |
|  |  | TraesCS2A02G522800 | F-box-like domain superfamily |
| 3064370 | 3A | TRITD3Av1G237380 | unknown |
|  |  | TraesCS3A02G552010LC | unknown |
| 1104851 | 3A | TRITD3Av1G008790 | Endo-1,4-beta-xylanase |
|  |  | TraesCS3B02G045600 | Glycoside hydrolase superfamily |
| 1090716 | 4A | TRITD4Av1G007190 | unknown |
|  |  | TraesCS4A02G018800LC^Ω^ | unknown |
| 2248753 | 4A | TRITD4Av1G007660^£^ | unknown |
|  |  | TraesCS4A02G024200^Ω^ | unknown |
| 1119379 | 4B | TRITD4Bv1G003710 ^Ω^ | unknown |
|  |  | TraesCS4B02G014700^£^ | UROD/MetE-like superfamily |
| 1092576 | 6A | TRITD6Av1G208790 ^Ω^ | Ankyrin repeat-containing protein, putative |
|  |  | TraesCS6A02G352300 | Ankyrin repeat-containing domain superfamily |
| 1078005^±^ | 6A | unknown | unknown |
|  |  | unknown | unknown |
| 10983799 | 6B | TRITD6Bv1G187920 | Xyloglucan fucosyltransferase |
|  |  | TraesCS6B02G351200 | Xyloglucan fucosyltransferase |
| 1074139 | 6B | TRITD6Bv1G218140 | Protein DMP |
|  |  | TraesCS6A02G388700 | Protein DMP |
|  |  |  |  |
|  |  |  |  |
| 1107872 | 2B | TRITD2Bv1G232900^£^ | SAUR-like auxin-responsive protein family |
|  |  | TRIAE_CS42_2BL_TGACv1_133071_AA0441210.1 ^Ω^ |  |
|  |  |  |  |
|  |  |  |  |
|  |  |  |  |
|  |  |  |  |
| 3064632 | 3B | TRITD3Bv1G021190 ^Ω^ | F-box family protein |
|  |  | TraesCS3B02G076400^£^  TraesCS3B02G093000LC^£^ | F-box family protein  Unknown |
| 4989018^†^ | 6A | TRITD6Av1G008950^£^ | unknown |
|  |  | TraesCS6A02G048200^£^ | Glycoside hydrolase superfamily |

^†^ Five SNPs of the nine SNPs identified as being in common among all eleven models were found within annotated high-confidence gene sequences in the reference genomes.

* Five SNPs identified at -log10 P ≥ 4.99 were found within annotated high-confidence gene sequences in the reference genomes.

^Ω^ Gene within 1000 bp upstream and downstream of the SNP position.

^£^ Closest gene to the SNP marker not within 1000 bp upstream and downstream.

^±^ no candidate gene was identified within a found thousands bp.
